# TGF-β1 dominates stromal fibroblast-mediated EMT via the FAP/VCAN axis in bladder cancer cells

**DOI:** 10.1186/s12967-023-04303-3

**Published:** 2023-07-17

**Authors:** Qinrong Ping, Chunhui Wang, Xin Cheng, Yiming Zhong, Ruping Yan, Meng Yang, Yunqiang Shi, Xiangmeng Li, Xiao Li, Wenwen Huang, Liqiong Wang, Xiaofang Bi, Libing Hu, Yang Yang, Yingbao Wang, Rui Gong, Jun Tan, Rui Li, Hui Li, Jian Li, Wenju Wang, Ruhong Li

**Affiliations:** 1 Department of Urology, Yan’an Hospital Affiliated to Kunming Medical University, Kunming, 650051 China; 2 Key Laboratory of Tumor Immunological Prevention and Treatment of Yunnan Provincial, Kunming, 650051 China; 3 Kunming Medical University, Kunming, 650051 China; 4 Department of Urology, The Second Affiliated Hospital of Kunming Medical University, Kunming, 650101 China; 5 Department of Pathology, Yan’an Hospital Affiliated to Kunming Medical University, Kunming, 650051 China; 6 Department of General Surgery, Yan’an Hospital Affiliated to Kunming Medical University, Kunming, 650051 China

**Keywords:** Bladder cancer, Stromal fibroblasts, Epithelial-mesenchymal transition, Transforming growth factor β1, Fibroblast activation protein, Versican

## Abstract

**Background:**

Bladder cancer is one of the most common malignant tumors of the urinary system and is associated with a poor prognosis once invasion and distant metastases occur. Epithelial-mesenchymal transition (EMT) drives metastasis and invasion in bladder cancer. Transforming growth factor β1 (TGF-β1) and stromal fibroblasts, especially cancer-associated fibroblasts (CAFs), are positive regulators of EMT in bladder cancer. However, it remains unclear how TGF-β1 mediates crosstalk between bladder cancer cells and CAFs and how it induces stromal fibroblast-mediated EMT in bladder cancer. We aimed to investigate the mechanism of TGF-β1 regulation of stromal fibroblast-mediated EMT in bladder cancer cells.

**Methods:**

Primary CAFs with high expression of fibroblast activation protein (FAP) were isolated from bladder cancer tissue samples. Subsequently, different conditioned media were used to stimulate the bladder cancer cell line T24 in a co-culture system. Gene set enrichment analysis, a human cytokine antibody array, and cytological assays were performed to investigate the mechanism of TGF-β1 regulation of stromal fibroblast-mediated EMT in bladder cancer cells.

**Results:**

Among the TGF-β family, TGF-β1 was the most highly expressed factor in bladder cancer tissue and primary stromal fibroblast supernatant. In the tumor microenvironment, TGF-β1 was mainly derived from stromal fibroblasts, especially CAFs. In stimulated bladder cells, stromal fibroblast-derived TGF-β1 promoted bladder cancer cell migration, invasion, and EMT. Furthermore, TGF-β1 promoted the activation of stromal fibroblasts, inducing CAF-like features, by upregulating FAP in primary normal fibroblasts and a normal fibroblast cell line. Stromal fibroblast-mediated EMT was induced in bladder cancer cells by TGF-β1/FAP. Versican (VCAN), a downstream molecule of FAP, plays an essential role in TGF-β1/FAP axis-induced EMT in bladder cancer cells. VCAN may also function through the PI3K/AKT1 signaling pathway.

**Conclusions:**

TGF-β1 is a critical mediator of crosstalk between stromal fibroblasts and bladder cancer cells. We revealed a new mechanism whereby TGF-β1 dominated stromal fibroblast-mediated EMT of bladder cancer cells via the FAP/VCAN axis and identified potential biomarkers (FAP, VCAN, N-cadherin, and Vimentin) of bladder cancer. These results enhance our understanding of bladder cancer invasion and metastasis and provide potential strategies for diagnosis, treatment, and prognosis.

**Supplementary Information:**

The online version contains supplementary material available at 10.1186/s12967-023-04303-3.

## Background

Bladder cancer (BLCA) is a common malignancy of the urinary system; it is ranked 12th for morbidity and 14th for mortality among systemic malignancies worldwide, and mainly occurs in the transitional epithelium [1]. The poor prognosis and challenging issues in BLCA research and treatment are mainly due to local invasion and distant metastasis [2]. Epithelial-mesenchymal transition (EMT) is a crucial mechanism underlying BLCA invasion and metastasis. Epithelial cancer cells acquire migratory and invasive capabilities through both EMT and crosstalk with the tumor microenvironment (TME), including stromal cells, immune cells, cytokines, chemokines, inflammatory mediators, and extracellular matrix (ECM) [3, 4].

Transforming growth factor β (TGF-β) signaling plays an irreplaceable role in BLCA EMT [5]. TGF-β present in the TME has many characteristics, such as abundant secretory sources, high concentrations, and pleiotropic effects. Earlier studies indicated that TGF-β produced by autocrine or paracrine factors mediates crosstalk between tumor cells and stromal fibroblasts to promote a fibrotic TME and local tissue fibrosis [6–8]. TGF-β1 is a key member of the TGF-β family, which induces normal fibroblasts (NFs) to transform into cancer-associated fibroblasts (CAFs). Mechanically, TGF-β1 drives stromal fibroblasts to exhibit tumor-promoting effects through canonical or noncanonical pathways by upregulating the expression of fibroblast activation protein (FAP) and α-smooth muscle actin (α-SMA/ACTA2) [9]. CAFs are "accomplice" cells in the TME with many characteristics including diverse origins, activation sophistication, heterogeneity and plasticity, and high abundance. CAFs contribute to the malignant behavior of multiple cancers through increased growth, proliferation, invasion, metastasis, immune escape, and therapy resistance by secreting various bioactive substances, including cytokines, growth factors, chemokines, and ECM proteins [10]. TGF-β1 and CAFs are both positive regulators of EMT in BLCA [11]. However, it remains unclear how TGF-β1 mediates crosstalk between BLCA cells and CAFs, and how it induces CAF-mediated EMT in BLCA.

In this study, we aimed to investigate the mechanism of TGF-β1 mediated crosstalk between BLCA cells and CAFs. Furthermore, we assessed how TGF-β1 regulated CAF-mediated EMT in BLCA.

## Methods

### Primary cell isolation and identification

A total of 18 samples were collected from patients who underwent radical or partial cystectomy at Yan’an Hospital Affiliated to Kunming Medical University from 2021 to 2022. Tissue specimen collection was approved by the ethics review committee (No. 2021-058-01), and all patients signed informed consent. Primary CAFs were isolated from BLCA tissues and primary NFs were isolated from normal bladder tissues (More than 5 cm from the tumor, the mucosa was smooth, and pathology confirmed normal tissue) via the collagenase digestion method (collagenase I, Solaibao, No. C8140, Beijing, China). Specifically, tissues were cleaned with PBS at 4 °C, cut into granules and digested with Dispase II solution (200 μg/ml) at 37 °C for 1 h. After washing with PBS, collagenase II (1.6 mg/ml) was added and incubated for 12 h. Cell suspensions were collected using a 70 μm cell filter. Cells were re-suspended in DMEM/F-12 medium after centrifugation and inoculated into T-25 bottles. Cell morphological observations and biomarker detection (FAP, FSP1, α-SMA, CD90, Vimentin) were used to identify CAFs and NFs.

### Cell culture

The BLCA cell line T24 (Procell Science & Technology Co., Ltd, Wuhan, China) underwent short tandem repeat testing for verification, and was cultured with RPMI 1640 medium (Gibco, No. C11875500BT, Shanghai, China) containing 10% fetal bovine serum (FBS) (Bio Channel, BC-SE-FBS07, Nanjing, China) and 1% penicillin/streptomycin (Biosharp, BL505, Hefei, China). The fibroblast cell line HFF (Cell Library of Typical Culture Preservation Committee of the Chinese Academy of Sciences) was cultured with high glucose DMEM medium (Biological Industries, No. 06-1055-57-1ACS, Shanghai, China) containing 10% FBS and 1% penicillin/streptomycin. Primary CAFs and NFs were cultured in DMEM/F12 medium (Biological Industries, No. 01-170-1A, Shanghai, China) containing 10% FBS and 1% penicillin/streptomycin. All cells were cultured at 37 °C in a CO_2_ incubator and passaged using EDTA-pancreatin digestion (Biosharp, No. BL512A, Hefei, China).

### Collection of conditioned medium

We selected approximately 1 × 10^6^ cells in the logarithmic growth phase and seeded them in T-25 culture flasks. Cells were cultured until confluence reached 80–90%. Cell supernatants were collected as corresponding conditioned medium (CM) for subsequent experiments. Supernatants of CAF, NF, HFF, ^OE^FAP-HFF (FAP overexpression), ^NC^FAP-HFF (FAP control), ^OE^VCAN-HFF (Versican overexpression), and ^NC^VCAN-HFF (VCAN control) cells were collected. In some cases, fibroblasts were pre-stimulated before co-culture assays. These cells were cultured until confluence reached 70–80%. Subsequently, recombinant human TGF-β1 (10 ng/ml, PeproTech, No. 100-21, USA) and/or TGF-β1 neutralizing antibody (0.15 μg/ml, Biolegend, No. 947303, Beijing, China) and/or control IgG (0.15 μg/ml, Biolegend, No. 400165, Beijing, China) was added to the culture medium to stimulate cells for 24 h. The supernatant was collected for the following experiments.

### Co-culture assay

T24 cells were selected in the logarithmic growth phase and treated according to the following steps: digestion, counting, plating, and attachment overnight. The culture medium was replaced with the corresponding CM. Cells were then collected for the following experiments.

### Quantitative real-time PCR (qRT-PCR)

Total RNA was extracted using Trizol^®^ Reagent (Life, No. 15596-018, USA). Reverse transcription was performed using the SureScript™ First-Strand cDNA Synthesis Kit (Genecopoeia, No. qp056, Guangzhou, China). mRNA expression levels of TGF-β1, FAP, VCAN, E-cadherin, N-cadherin, Vimentin, PI3K, and AKT1 were detected by qRT-PCR using BlazeTaq™ SYBR^®^Green qPCR Mix 2.0 (Genecopoeia, No. qp056, Guangzhou, China). GAPDH was selected as an internal control. Primer sequences (Tsingke Biotechnology, Beijing, China) are shown in Additional file 1.

### Western blotting (WB)

RIPA lysis solution (Beyotime, No. P0013B, Shanghai, China) with 1% PMSF (APEXBIO, No. K1007, Houston, USA) was used for cell lysis. The extracted protein concentration was determined using the BCA Quantitative Detection kit (Beyotime, No. P0009-1, Shanghai, China). Proteins were separated by electrophoresis on SDS-PAGE gels and blots were transferred to polyvinylidene difluoride (PVDF) membranes (Millipore, K2MA8350E). Membranes were washed once in Tris-buffered saline with Tween (TBST) (Solaibao, Beijing, China), following blocking in 5% skimmed milk in TBST (BD, No. 2271470) for 30 min at room temperature. Membranes were incubated with primary antibodies (diluted in 5% skimmed milk; Additional file 2) overnight at 4 °C, then for 1 h at room temperature the following day. Membranes were washed three times with TBST (5 min/wash) and incubated with diluted secondary antibodies on a shaker for 30 min. The coordinated ECL solution was added to the membrane (Immobilon Western HRP Substrate Luminol Reagent, Affinity, No. KF001) and a gel imager was used to capture the image. Relative protein expression was calculated with ImageJ.

### Dot blot

Bladder cancer tissue and normal bladder tissue were collected from patients. We used the same method and procedure for sample preparation as we used for western blotting. Following sample preparation, 2 μl sample was placed on a PVDF membrane, fixed at 37 °C for 30 min, sealed for one hour, primary antibody binding at room temperature for 30 min, washed, secondary antibody binding at room temperature for 30 min, washed, exposed, and analyzed statistically by ImageJ.

### Enzyme-linked immunosorbent assay (ELISA)

VCAN, TGF-β1, TGF-β2, and TGF-β3 protein in cell supernatants were detected with the ELISA kits (Additional file 3).

### Immunofluorescence

The 24-well plate of cell crawling slides was immersed in PBS (Solarbio, No. P1003, Beijing, China) for 3 min twice before fixing for 1 h with 4% paraformaldehyde and storing at 4 °C. Citrate repair solution pH 6.0 (ZSGB-Bio, No. ZLI-9065, Beijing, China) was added following immersion, heating, natural cooling, and immersion in PBS. Next, 0.2% Triton X-100 was applied at room temperature for 20 min before immersing in PBS. Slides were blocked at 37 °C for 30 min with 5% bovine serum albumin (BSA) and then incubated in diluted primary antibody (Additional file 4) overnight at 4 °C. Following incubation for 30 min at 37 °C, slides were immersed in PBS, and then incubated in diluted fluorescent secondary antibodies at 37 °C for 30 min. After immersion in PBS, DAPI (Solarbio, NO. D8200, Beijing, China) was applied for 5–7 min at room temperature in the dark, before re-immersing the slides in PBS. A tablet sealing liquid containing an anti-fluorescence quencher was used to seal crawling slides. Images were captured with a fluorescence microscope. The positive rate of the images was calculated using Image-Pro Plus.

### Wound healing assay

T24 cells in the logarithmic growth phase were digested with trypsin (Biosharp, No. BL512A) into single-cell suspensions and seeded at 6 × 10^5^ cells/well in 2 ml serum-free medium in 6-well plates. Following 24 h of culture, 3–5 lines/well were drawn. Cells were washed excessively three times with PBS and each group received their CM. Photos were taken at 0 h and 12 h.

### Transwell assay

Matrigel (Corning, No. 356234, USA) was placed at 4 °C overnight. The following day, Matrigel was diluted with serum-free 1640 medium to 300 μg/ml and 100 μl was spread evenly on the polyester (PET) membrane in the cell culture tank placed in a 24-well plate. In the upper chamber, the medium was absorbed after 30 min at 37 °C. T24 cells in the logarithmic growth phase were suspended in 200 ul serum-free medium and added to the upper chamber at 1 × 10^4^ cells/well. As needed, complete or CM was added to the lower chamber until it touched the bottom of the 8-μm Transwell chamber (Corning, No. 3422, USA). Cells were cultured for 24 h, washed twice with PBS, fixed with methanol for 10 min, dried, and stained with 0.5% crystal violet solution (Solarbio, No. C8470, Beijing, China) for 20 min. The PET film was wiped with a cotton swab and rinsed with PBS until the background was clear. Slices were sealed, and cells were counted.

### Human cytokine antibody array

Supernatant was collected from six cell groups and sent to RayBiotech (Guangzhou, China) for human cytokine antibody array detection. We set the six samples into three control groups (P5 NF-CM vs. P3 CAF-CM, P5 NF-CM vs. P6 CAF-CM, ^NC^FAP-NF-CM vs. ^OE^FAP-NF-CM) and compared the differential factors in supernatants within each group. Cell passage was determined by growth state. Differential proteins were screened in the three control groups, and the intersection among the three control groups was used to guide the next study.

### Immunochemistry

Immunohistochemistry was performed as follows: preparing paraffin sections, baking (64 °C, 30 min), dewaxing, hydration, antigen repair (citrate repair solution pH 6.0, ZSGB-Bio, No. ZLI-9065, Beijing, China), blocking (H_2_O_2_), incubation with primary antibody (4 ℃, overnight) (Additional file 5), rewarming (37 ℃, 30 min), drop addition of reaction enhancer solution (37 ℃, 20 min), incubation with secondary antibody (37 ℃, 30 min), DAB chromogenic kit (DAB chromogenic kit, ZSGB-Bio, No. ZLI-9019, Beijing, China), hematoxylin counterstaining (Solarbio, No. H8070, Beijing, China), dehydration, transparency, sealing (Solarbio, No. NO. G8590, Beijing, China), image acquisition, and analysis.

### Subcutaneous xenograft model

FAP was overexpressed and knocked down in HFF cells. To establish a subcutaneous xenograft model, fibroblasts (4 × 10^6^ cells) and T24 cells (1 × 10^6^ cells) were mixed and co-injected subcutaneously into nude mice. A total of seven groups were created based on the type of fibroblasts (^OE^FAP-HFF + T24, ^OE^NC-HFF + T24, ^sh^FAP-HFF + T24, ^sh^NC-HFF + T24, NF + T24, and CAF + T24). Each group had five mice. The tumor formation was recorded, and the nude mice were killed on the 24th day. Molecule expression in tumor tissues was detected by qRT-PCT, WB, immunohistochemistry, and immunofluorescence.

### RNA sequencing and bioinformatics analysis

FPKM (Fragments per Kilobase per M letters) RNAseq data on BLCA were downloaded from the TCGA database (https://portal.gdc.cancer.gov/) database, while TPM (transcripts per million reads) format data were downloaded from GTEx normal tissues and TCGA BLCA samples (43 samples, including nine GTEx normal tissues, 407 cancers, and 19 paracancerous tissues); unified handling was performed by UCSC XENA (https://xenabrowser.net/datapages/) by the Toil process [12]. Prognostic data were derived from a Cell article [13]. FPKM and TPM RNAseq data were log2 transformed. Prognostic data were converted from FPKM to TPM format and log2 transformed. R software (version 3.6.3, Statistical Analysis and Visualization) was used for analysis, including “ggplot2” (version 3.3.3, for visualization), “survminer” (version 0.4.9, for visualization), “survival” (version 3.2-10, for statistical analysis of survival data), and “clusterProfiler” (version 3.14.3, for analysis of selected data). We analyzed TGFB1 [ENSG00000105329], TGFB2 [ENSG00000092969], TGFB3 [ENSG00000119699], FAP [ENSG00000078098], VCAN [ENSG00000038427], CDH1/E-cadherin [ENSG00000039068], CDH2/N-cadherin [ENSG00000170558], and VIM [ENSG00000026025].

Firstly, we compared FAP expression in cancerous and paracancerous tissues. Secondly, single-gene differential expression analysis was performed on FAP expression. Extracted TCGA-BLCA RNAseq data and clinical data from the TCGA database. The DESeq2 package was then used to screen differentially expressed molecules between high and low FAP expression groups. Thirdly, we sorted the intersection result of the human cytokine antibody array according to the differential expression level in screened protein-coding genes from FAP single-gene differential expression analysis. Next, we performed Gene Ontology and Kyoto Encyclopedia of Genes and Genomes (GO/KEGG) enrichment analyses for protein-coding genes with logFC > 2 and Padj > 0.05. All protein-coding genes from the FAP single-gene differential expression analysis were processed for gene set enrichment analysis (GSEA) in the "HALLMARK_EPITHELIAL_MESENCHYMAL_TRANSITION" gene sets. Next, we compared the expression of VCAN in cancerous and paracancerous tissues (paired samples) and verified the correlation between FAP and VCAN. Furthermore, co-expression analysis of FAP and molecular correlation analysis of TGF-β1, VCAN, E-cadherin, N-cadherin, and Vimentin were performed. The clinical correlation between TGF-β1, FAP, VCAN, E-cadherin, N-cadherin, Vimentin, and BLCA was further verified. Finally, FAP, VCAN, N-cadherin, and Vimentin were selected for prognosis and survival analysis.

### Statistical analysis

Statistics were performed using GraphPad Prism 6.0. A t-test and nonparametric test were used to analyze expression differences and P < 0.05 was considered statistically significant. Correlation bar statistics were drawn with GraphPad Prism 6.0 showing mean differences of standard errors. Significance identification: ^NS^P ≥ 0.05, *P < 0.05, **P < 0.01, ***P < 0.001, ****P < 0.0001.

## Results

### Isolation and identification of primary CAFs and NFs

**Fig. 1:**
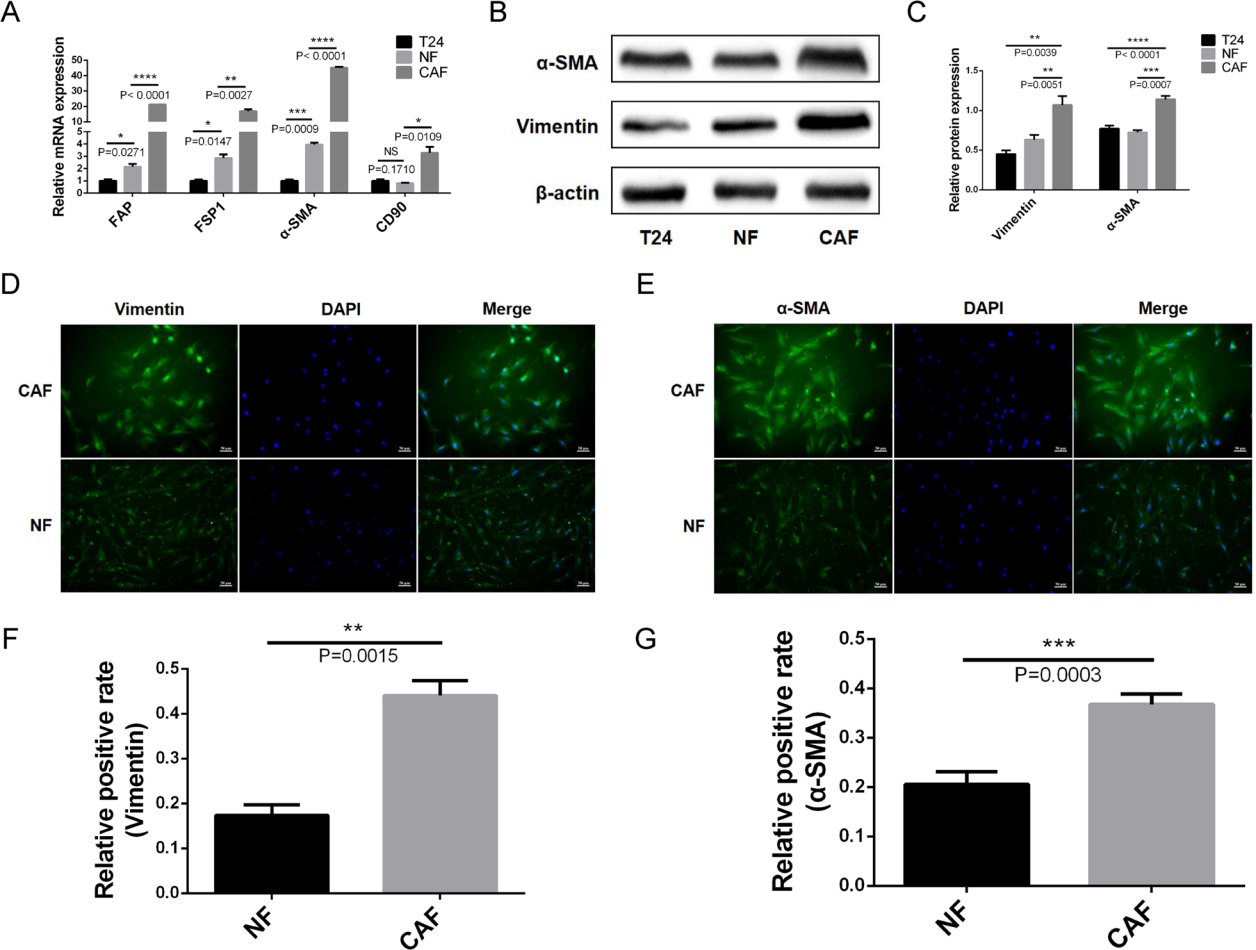
Identification of primary CAFs and NFs. **A** qRT-PCR results shows that the mRNA expression levels of FAP, FSP1, α-SMA, and CD90 in CAFs are significantly higher than those in NFs and T24 cells. **B**, **C** WB verified that the protein expression levels of α-SMA and Vimentin in CAFs are significantly higher than those in NFs and T24 cells. **D**–**G** Immunofluorescence staining shows that CAFs and NFs have an elongated spindle morphology, and CAFs express more Vimentin and α-SMA than NFs

We successfully isolated CAFs and NFs from BLCA and normal bladder tissues by collagenase digestion. CAFs and NFs were identified by morphological observation and the expression of FAP, FSP1, α-SMA, CD90, and Vimentin. mRNA expression levels of FAP, FSP1, α-SMA, and CD90 were significantly higher in CAFs than in NFs and T24 cells (Fig. 1A). WB results further verified that the protein expression levels of FAP, α-SMA, and Vimentin were significantly higher in CAFs than in NFs and T24 cells (Fig. 1B, C). Immunofluorescence staining showed that CAFs and NFs had an elongated spindle morphology; CAFs expressed more Vimentin and α-SMA than NFs (Fig. 1D–G).

### TGF-β1 in the TME was mainly derived from stromal fibroblasts, which may play a key role in BLCA cell migration

**Fig. 2:**
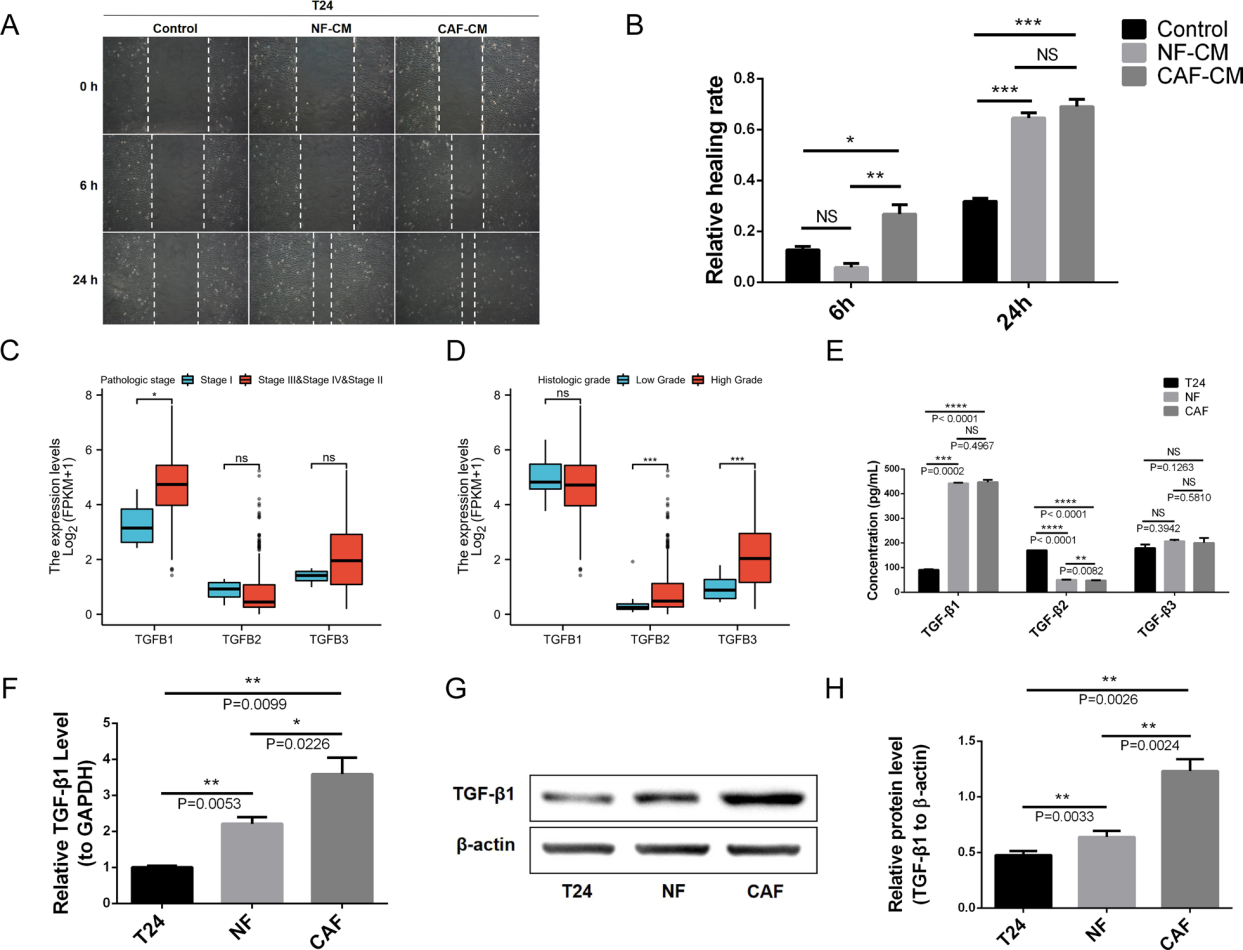
Source and expression of TGF-β1 in the tumor microenvironment. **A**, **B** CAF-CM and NF-CM promotes BLCA cell migration in a wound-healing assay. **C** The expression level of TGF-β1 in different pathological stages of BLCA is higher than that of TGF-β2 and TGF-β3. **D** The expression level of TGF-β1 in different histological grades of BLCA is higher than that of TGF-β2 and TGF-β3. **E** ELISA shows that the concentration of TGF-β1 in primary CAF and NF supernatant is significantly higher than TGF-β2 and TGF-β3, and that TGF-β1 derived from stromal fibroblasts is considerably higher than tumor cells. **F** qRT-PCR indicates that the mRNA expression levels of TGF-β1 in CAFs and NFs are significantly higher than in T24 cells. **G**, **H** WB shows that the protein expression levels of TGF-β1 in CAFs and NFs are notably higher than in T24 cells. **F**–**H** CAFs express the highest level of TGF-β1 compared to the other cell types

T24 cells were stimulated with CAF-CM and NF-CM in a co-culture system; both promoted migration in a wound healing assay (Fig. 2A, B).

Although both TGF-β signaling and CAFs promote EMT in various cancers [5, 14], it is unclear which of the TGF-β family members (TGF-β1, TGF-β2, and TGF-β3) are responsible in BLCA. Therefore, we analyzed the expression levels of the three most common members of the TGF-β family in BLCA by bioinformatics analysis. The expression level of TGF-β1 in BLCA tissues was higher than TGF-β2 and TGF-β3 (Fig. 2C, D). In addition, ELISAs showed that the concentration of TGF-β1 in primary CAF and NF supernatant was significantly higher than TGF-β2 and TGF-β3 (Fig. 2E). Specifically, both tumor cells and stromal fibroblasts (CAFs and NFs) could secrete TGF-β1; however, TGF-β1 secreted by the stromal fibroblasts was significantly higher than tumor cells (Fig. 2E), suggesting that TGF-β1 in the TME is mainly secreted by stromal fibroblasts.

Therefore, we selected TGF-β1 for further studies and verified its expression in T24, NF, and CAF cells. mRNA expression levels of TGF-β1 in CAFs and NFs were significantly higher than in T24 cells (Fig. 2F). WB showed that the protein expression levels of TGF-β1 in CAFs and NFs were significantly higher than in T24 cells (Fig. 2G, H). Importantly, CAFs expressed the highest level of TGF-β1 compared to the other cell types (Fig. 2F–H). Therefore, TGF-β1 in the TME is mainly derived from stromal fibroblasts, especially from CAFs, which may play a key role in BLCA cell migration.

### TGF-β1 derived from stromal fibroblasts promotes BLCA cell migration, invasion, and EMT

**Fig. 3:**
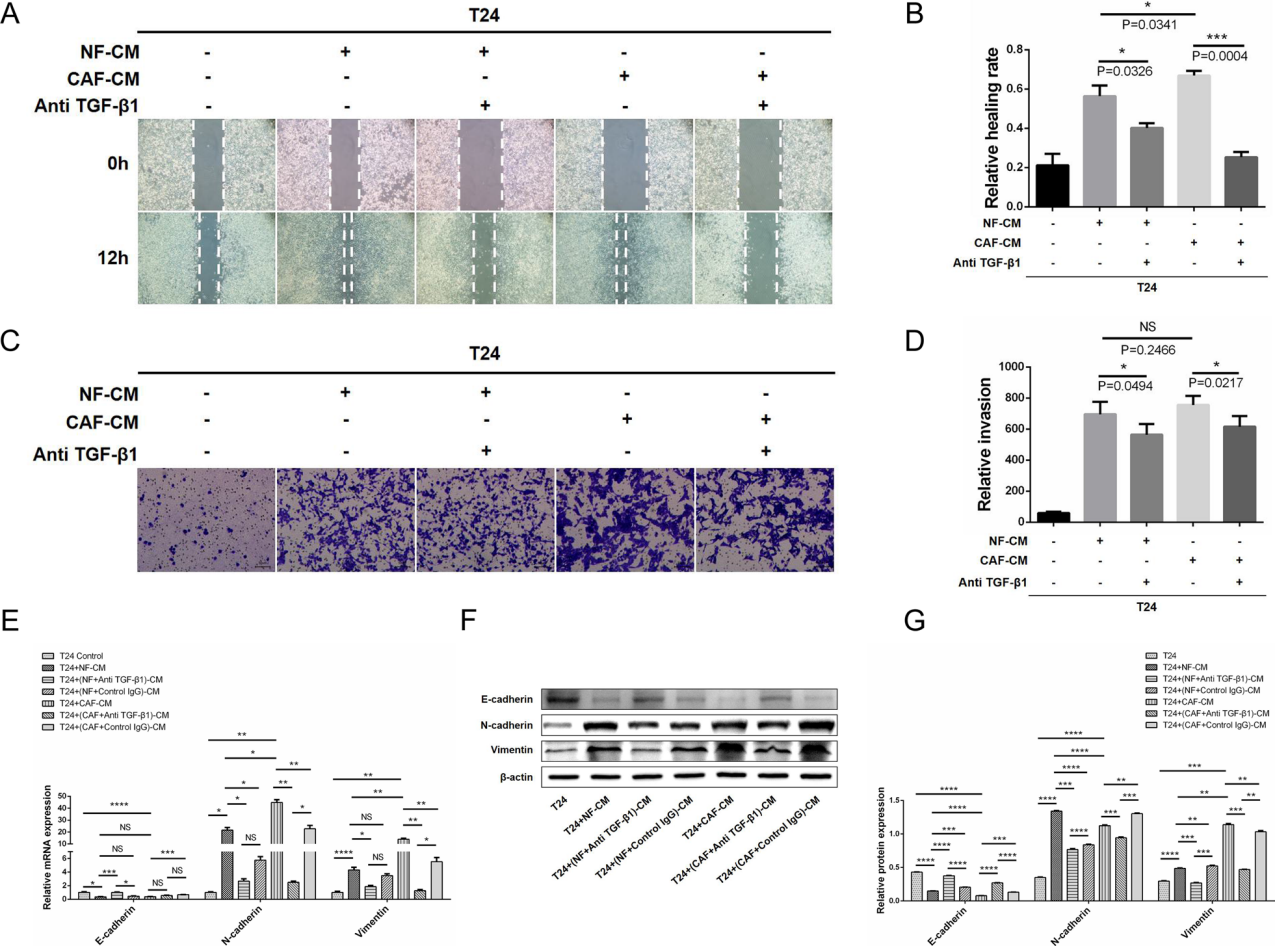
TGF-β1 derived from stromal fibroblasts promotes BLCA cell migration, invasion, and EMT. **A**, **B** A wound healing assay shows that CAF-CM and NF-CM significantly enhance the migration ability of T24 cells; TGF-β1 neutralizing antibody attenuates this pro-migration effect. **C**, **D** A transwell assay suggests that CAF-CM and NF-CM markedly improve the invasive ability of T24 cells; TGF-β1 neutralizing antibody effectively reduced this pro-invasion effect. qRT-PCR (**E**) and WB (**F**, **G**) show that CAF-CM and NF-CM induce EMT in BLCA cells by downregulating the expression of E-cadherin and upregulating the expression of N-cadherin and Vimentin. **E**–**G** The TGF-β1 neutralizing antibody significantly attenuated these pro-EMT effects

We added TGF-β1 neutralizing antibodies to the co-culture system of CAF-CM and NF-CM induced T24 cells; CAF-CM and NF-CM significantly enhanced T24 cell migration and invasion in wound healing assays (Fig. 3A, B) and transwell assays (Fig. 3C, D), respectively. In contrast, the TGF-β1 neutralizing antibody significantly abrogated these pro-migration and pro-invasion effects. Furthermore, the expression of epithelial biomarkers E-cadherin and interstitial biomarkers N-cadherin and Vimentin in co-cultured T24 cells was evaluated by qRT-PCR and WB. CAF-CM and NF-CM promoted EMT by downregulating E-cadherin expression and upregulating N-cadherin and Vimentin expression (Fig. 3E–G). Similarly, TGF-β1 neutralizing antibody attenuated the EMT-promoting effects of CAF-CM and NF-CM in BLCA cells. Briefly, TGF-β1 derived from stromal fibroblasts promotes migration, invasion, and EMT of BLCA cells, which are attenuated by antagonizing TGF-β1.

### TGF-β1 promotes stromal fibroblasts into an activated state with CAF-like features by upregulating FAP

**Fig. 4:**
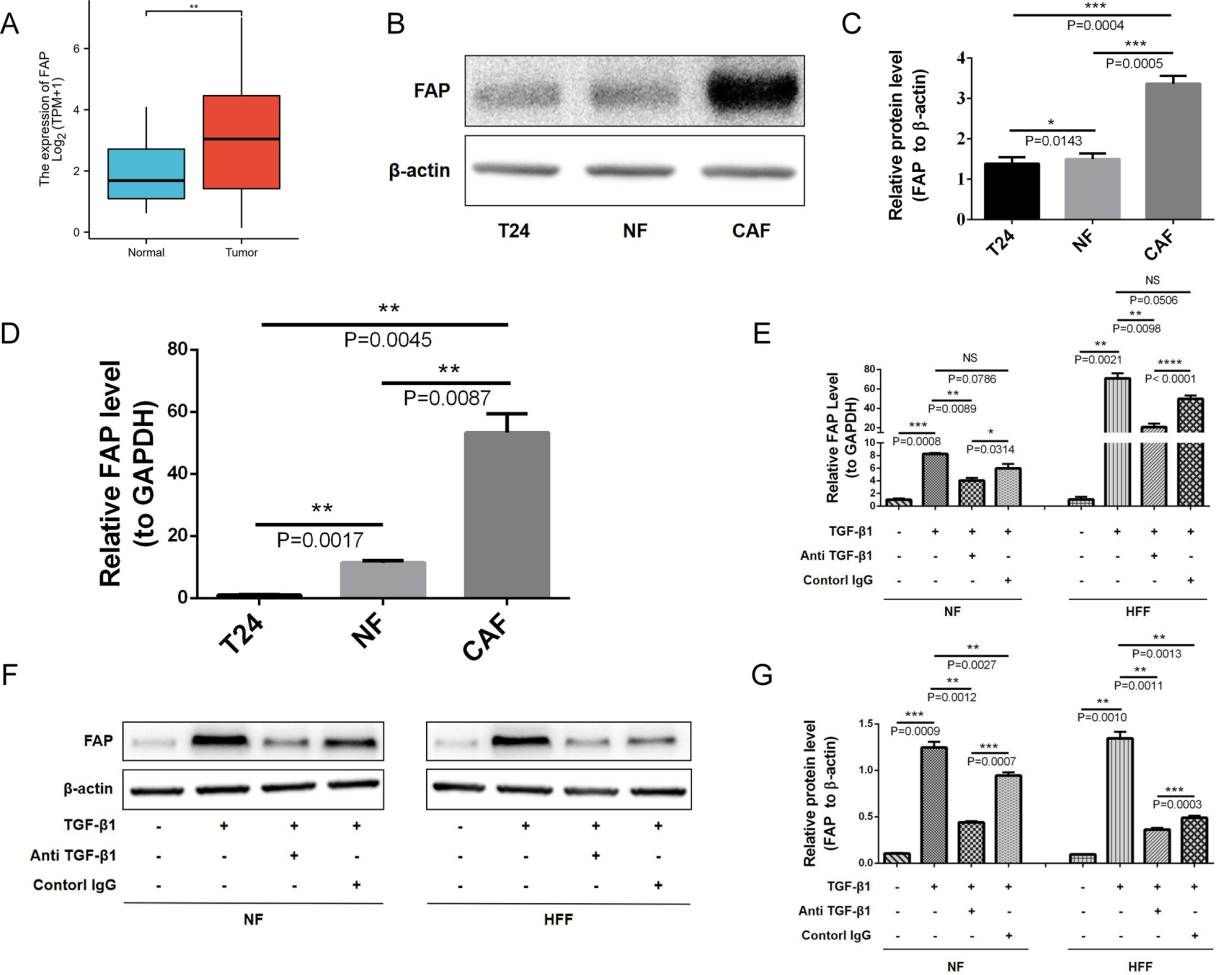
TGF-β1 promotes stromal fibroblasts into an activated state with CAF-like features by upregulating FAP. **A** Bioinformatics analysis revealed that BLCA tissues express higher levels of FAP than adjacent normal tissues. **B**, **C** WB shows that CAFs express more FAP than NFs and T24 cells. **D** FAP mRNA expression is significantly higher in CAFs than in NFs and T24 cells, based on qRT-PCR. **E** qRT-PCR shows that TGF-β1 upregulates FAP expression levels in NFs and HFFs, and that neutralizing antibodies could reverse this. **F**, **G** WB shows that TGF-β1 upregulates the protein expression level of FAP in NFs and HFFs, and neutralizing antibodies could reverse this

Besides being a biomarker for CAFs, FAP is also a biomarker for stromal fibroblasts activation. However, there is still much to learn about the relationship between TGF-β1 and FAP, and how these two molecules regulate stromal fibroblast-mediated function in BLCA. Bioinformatics analysis revealed that BLCA tissues expressed higher levels of FAP than adjacent normal tissues (Fig. 4A). Both WB (Fig. 4B, C) and qRT-PCR (Fig. 4D) demonstrated that CAFs expressed more FAP than NFs and T24 cells. These results demonstrate the significant upregulation of FAP expression in BLCA tissues and primary CAFs.

To further verify whether TGF-β1 in the TME is correlated with NF activation and increased expression of FAP, we used exogenous TGF-β1 to induce primary NFs and HFFs. TGF-β1 upregulated FAP mRNA levels in NFs and HFFs, whereas TGF-β1 neutralizing antibody reverted this effect (Fig. 4E). Similarly, WB showed that TGF-β1 positively influenced the expression of FAP at the protein level (Fig. 4F, G). Therefore, TGF-β1 promotes stromal fibroblasts into an activated state with CAF-like features by upregulating FAP.

### TGF-β1 promotes stromal fibroblast-mediated EMT in BLCA cells by upregulating FAP

**Fig. 5:**
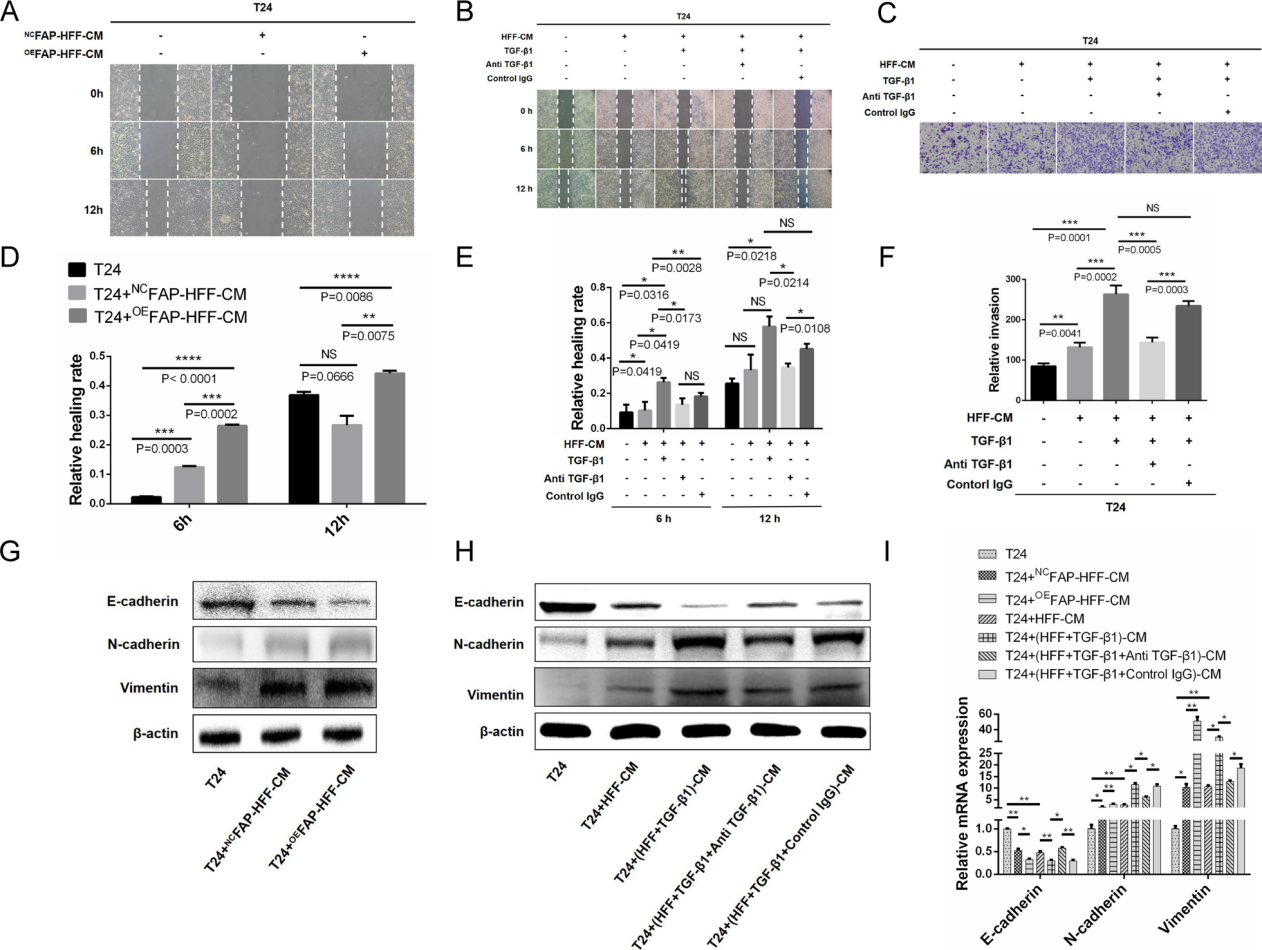
TGF-β1 promotes fibroblast-mediated EMT in BLCA cells by upregulating FAP. **A**, **D** A wound healing assay demonstrates that ^OE^FAP-HFF-CM enhances the migration of T24 cells. **B**, **E** A wound healing assay shows that HFF-CM promotes T24 cell migration, and TGF-β1-induced HFF-CM amplifies the pro-migration effects, while TGF-β1 neutralizing antibody reverses this amplified pro-migration effect. **C**, **F** A transwell assay demonstrates that HFF-CM promotes T24 cell invasion, and TGF-β1-induced HFF-CM enhances the pro-invasion effects, while TGF-β1 neutralizing antibody reverses this enhanced pro-invasion effects. **G**, **H** WB and (**I**) qRT-PCR show that HFF-CM (stimulated or not stimulated) has a pro-EMT effect, with downregulated E-cadherin and upregulated N-cadherin and Vimentin. The pro-EMT effects of HFF-CM were significantly amplified after TGF-β1 induction or overexpression FAP, while the TGF-β1 neutralizing antibody reverses the pro-EMT effects of TGF-β1 induction

FAP is a prominent marker of activated fibroblasts [9]. Whether FAP could act as a downstream molecule of TGF-β1 to promote EMT in BLCA cells remains to be discovered. Therefore, we established a fibroblast cell line with FAP overexpression, ^OE^FAP-HFF, and its control, ^NC^FAP-HFF, and verified that the expression of FAP in ^OE^FAP-HFF was significantly higher than that in ^NC^FAP-HFF and T24 cells through qRT-PCR and WB (Additional file 6A–C). Next, ^OE^FAP-HFF and ^NC^FAP-HFF CM was collected and co-cultured with T24 cells. A wound healing assay demonstrated that ^OE^FAP-HFF-CM could promote T24 cell migration (Fig. 5A, D). To further demonstrate the effects of fibroblasts and the TGF-β1/FAP axis on migration, invasion, and EMT of BLCA cells, we collected CM from HFF cells and exogenous factor-induced HFF cells after 24 h induction with exogenous TGF-β1 and/or TGF-β1 neutralizing antibody and/or control IgG. In wound healing assays (Fig. 5B, E) and transwell assays (Fig. 5C, F), HFF-CM promoted T24 cell migration and invasion. Increasingly, TGF-β1-induced HFF-CM could amplify the pro-migration and pro-invasion effects. In turn, TGF-β1 neutralizing antibodies could significantly diminish the effects of TGF-β1. Finally, we used WB (Fig. 5G, H Additional file 6D, E) and qRT-PCR (Fig. 5I) to detect the expression of EMT markers in T24 cells in different co-culture systems and found that HFF-CM (stimulated or not stimulated) had a pro-EMT effect, with downregulated E-cadherin and upregulated N-cadherin and Vimentin. It is worth noting that ^OE^FAP-HFF-CM and TGF-β1-induced HFF-CM had an enhanced pro-EMT effect. Conversely, TGF-β1 neutralizing antibody reversed the enhanced pro-EMT effects of TGF-β1. Thus, TGF-β1 promotes fibroblast-mediated EMT in BLCA cells by upregulating FAP.

### Overexpressing FAP induces stromal fibroblasts to secrete more VCAN

**Fig. 6:**
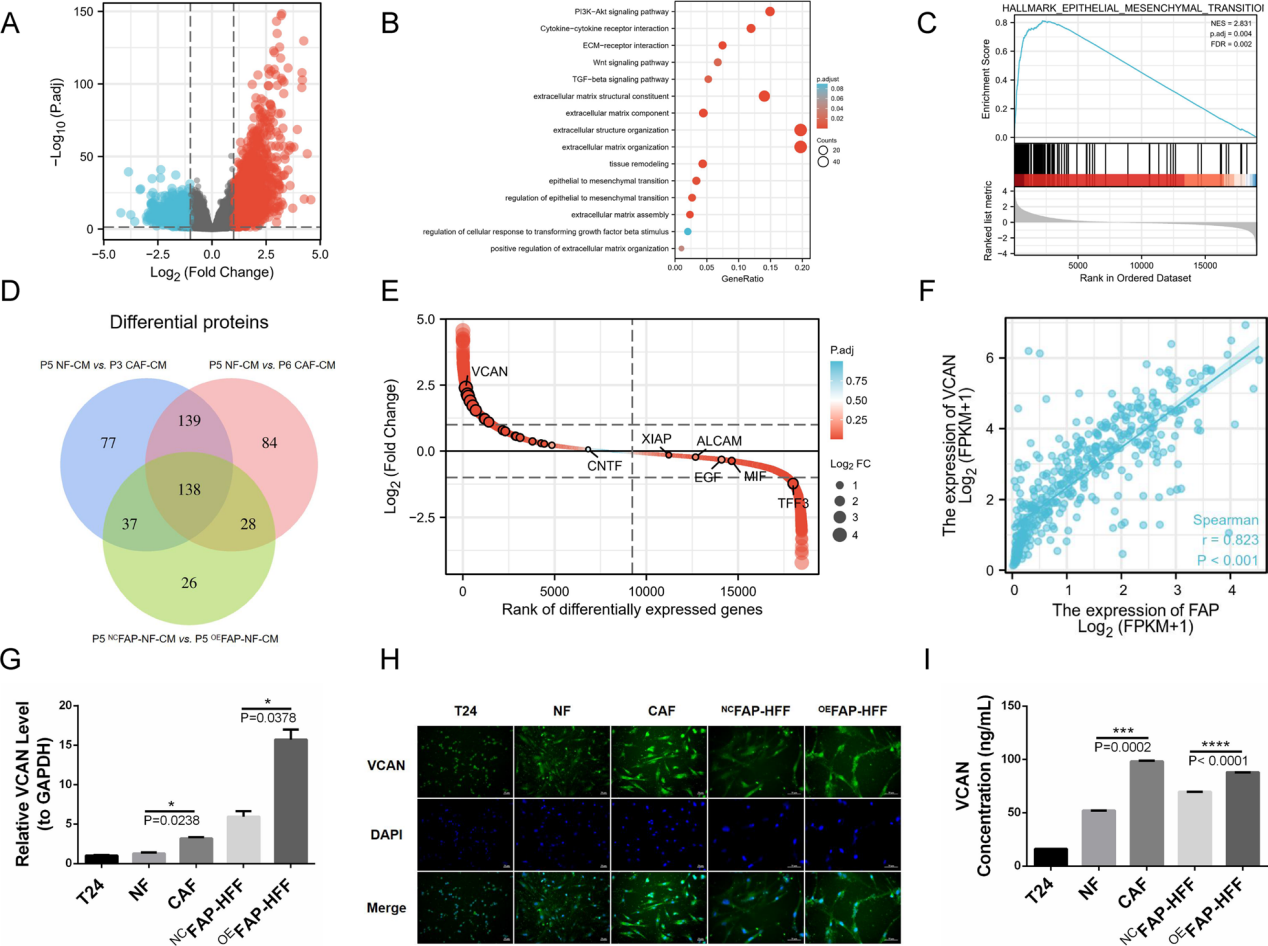
Overexpressing FAP induces stromal fibroblasts to secrete more VCAN. **A** Single-gene differential expression analysis of FAP in BLCA is presented in the volcano map. **B** GO/KEGG enrichment analyses showed that the PI3K/AKT signaling pathway was significantly enriched in protein-coding genes with logFC > 2 and Padj > 0.05. **C** GSEA enrichment analysis based on EMT shows that differential protein-coding genes of FAP correlate positively with EMT characteristics of BLCA. **D** A total of 138 differentially expressed proteins were identified from the intersection of the three control groups. **E** VCAN is the most highly expressed protein-coding gene among the differential protein-coding genes related to FAP. **F** FAP expression correlates positively with VCAN in a molecular correlation. **G** qRT-PCR demonstrates that the mRNA expression level of VCAN in CAFs is significantly higher than in NFs, and in ^OE^FAP-HFFs it is significantly higher than in ^NC^FAP-HFFs. **H** Immunofluorescence shows that the fluorescence intensity of VCAN in CAFs is notably brighter than in NFs, and in ^OE^FAP-HFFs it is notably brighter than in ^NC^FAP-HFFs. **I** ELISA revealed that the concentration of VCAN in CAF supernatant is considerably higher than in NF supernatant, and in ^OE^FAP-HFF supernatant is considerably higher than in ^NC^FAP-HFF supernatant

To explore whether downstream factors of FAP are related to EMT of BLCA cells, we performed single-gene differential expression analysis of FAP in BLCA and presented the results in the volcano map (Fig. 6A). Protein-coding genes with logFC > 2 and Padj > 0.05 were screened and used the GO/KEGG enrichment analyses, which showed that the PI3K/AKT signaling pathway was significantly enriched (Fig. 6B). All protein-coding genes were screened out to follow the GSEA enrichment analysis based on EMT, which revealed that differential protein-coding genes of FAP positively correlated with EMT characteristics of BLCA (Fig. 6C). Next, we compared the CM from six cell samples in three control groups (P5 NF-CM vs. P3 CAF-CM, P5 NF-CM vs. P6 CAF-CM, ^NC^FAP-NF-CM vs. ^OE^FAP-NF-CM) using a human cytokine antibody array (Fig. 6D). A total of 138 differentially expressed proteins were identified from the intersection of the three control groups and were sorted among the differential protein-coding genes related to FAP; VCAN was the most significant (Fig. 6E). Hence, we speculated that VCAN is downstream of FAP. Clinically, the expression of VCAN in BLCA tissues was significantly higher than in adjacent normal tissues (Additional file 7A). A molecular correlation between FAP and VCAN showed that FAP expression was positively correlated with VCAN (Fig. 6F). The mRNA expression level of VCAN in CAFs was significantly higher than in NFs, and in ^OE^FAP-HFF it was significantly higher than in ^NC^FAP-HFF (Fig. 6G). Immunofluorescence showed that the fluorescence intensity of VCAN in CAFs was notably brighter than in NFs, and in ^OE^FAP-HFF it was notably brighter than in ^NC^FAP-HFF (Fig. 6H; Additional file 7B). An ELISA revealed that the concentration of VCAN in CAF supernatant was considerably higher than in NF supernatant, and in ^OE^FAP-HFF supernatant it was considerably higher than in ^NC^FAP-HFF supernatant (Fig. 6I). Therefore, VCAN is a downstream molecule of FAP, and its expression and secretion were dramatically upregulated in stromal fibroblasts with high expression levels of FAP.

### TGF-β1/FAP axis promotes stromal fibroblast-mediated EMT in BLCA cells by upregulating VCAN

**Fig. 7:**
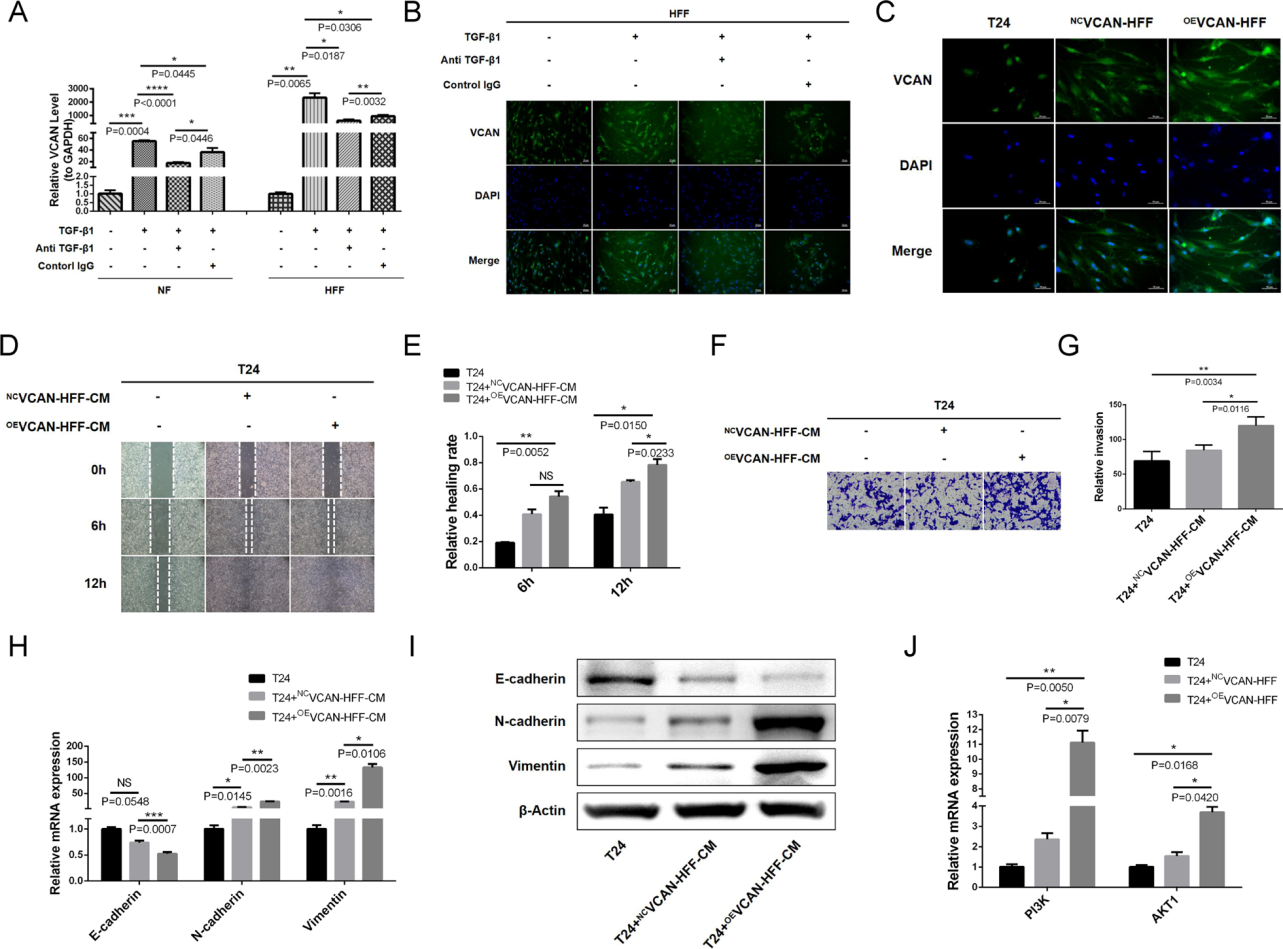
TGF-β1/FAP axis promotes fibroblast-mediated EMT in BLCA cells by upregulating VCAN. **A** qRT-PCR shows that TGF-β1 upregulates the mRNA expression level of VCAN in NF and HFF; TGF-β1 neutralizing antibody reverses the upregulation effect of TGF-β1 on VCAN. **B** Immunofluorescence shows that the fluorescence intensity of VCAN in HFF is markedly enhanced under the induction of TGF-β1; TGF-β1 neutralizing antibody significantly reduces this enhanced fluorescence intensity. **C** Immunofluorescence reveals that the VCAN fluorescence intensity in ^OE^VCAN-HFF is notably brighter than in ^NC^VCAN-HFF and T24 cells. **D**, **E** A wound healing assay shows that ^OE^VCAN-HFF-CM promotes T24 cell migration. **F**, **G** A transwell assay suggests that ^OE^VCAN-HFF-CM promotes T24 cell invasion. **H** qRT-PCR and (**I**) WB show that ^OE^VCAN-HFF-CM can downregulate E-cadherin as well as upregulate N-cadherin and Vimentin. **J** qRT-PCR show that ^OE^VCAN-HFF-CM increase PI3K and AKT1 mRNA levels in T24 cells

To further explore the communication between TGF-β1/FAP/VCAN axis in fibroblasts and EMT in BLCA cells, we used exogenous TGF-β1 to induce NFs and HFFs and found that the expression trend of VCAN was consistent with FAP. qRT-PCR showed that TGF-β1 could upregulate the mRNA expression level of VCAN in NFs and HFFs, and TGF-β1 neutralizing antibody could reverse this effect (Fig. 7A). Immunofluorescence showed that the fluorescence intensity of VCAN in HFFs was markedly enhanced under the induction of TGF-β1, and TGF-β1 neutralizing antibody significantly reduced this effect (Fig. 7B, Additional file 8A).

The above studies have shown that the TGF-β1/FAP axis promotes EMT in BLCA cells and regulates VCAN in fibroblasts. However, the relationship between VCAN and EMT remains unclear. We established a fibroblast cell line with VCAN overexpression, ^OE^VCAN-HFF, and its control, ^NC^VCAN-HFF, and verified that the expression of VCAN in ^OE^VCAN-HFF was significantly higher than in ^NC^VCAN-HFF and T24 cells by qRT-PCR (Additional file 8B). Immunofluorescence revealed that the VCAN fluorescence intensity in ^OE^VCAN-HFF was notably brighter than in ^NC^VCAN-HFF and T24 cells (Fig. 7C; Additional file 8C). ELISA showed that the concentration of VCAN in the ^OE^VCAN-HFF supernatant was significantly higher than in ^NC^VCAN-HFF and T24 cells (Additional file 8D). We co-cultured CM from ^OE^VCAN-HFF or ^NC^VCAN-HFF with T24 cells. The wound healing assay showed that ^OE^VCAN-HFF-CM promoted T24 cell migration (Fig. 7D, E). Consistently, the transwell assay suggested that ^OE^VCAN-HFF-CM promoted T24 cell invasion (Fig. 7F, G). Following the co-culture system, qRT-PCR (Fig. 7H) and WB (Fig. 7I, Additional file 8E) were used to detect the expression of EMT markers in T24 cells; ^OE^VCAN-HFF-CM downregulated E-cadherin and upregulated N-cadherin and Vimentin. ^OE^VCAN-HFF showed similar EMT-promoting effects as primary CAFs and ^OE^FAP-HFFs. Since we found that the PI3K/AKT signaling pathway was significantly enriched by GO/KEGG analysis (Fig. 6B). We examined the correlation between VCAN and the PI3K/AKT signaling pathway using qRT-PCR and found that ^OE^VCAN-HFF-CM could significantly increase PI3K and AKT1 mRNA levels in T24 cells (Fig. 7J). Therefore, the TGF-β1/FAP axis promotes fibroblast-mediated EMT in BLCA cells by upregulating VCAN and VCAN may play its role through the PI3K/AKT signaling pathway in T24 cells.

### *TGF-β1/FAP/VCAN axis promotes stromal fibroblast-mediated EMT in bladder cancer *in vivo

**Fig. 8:**
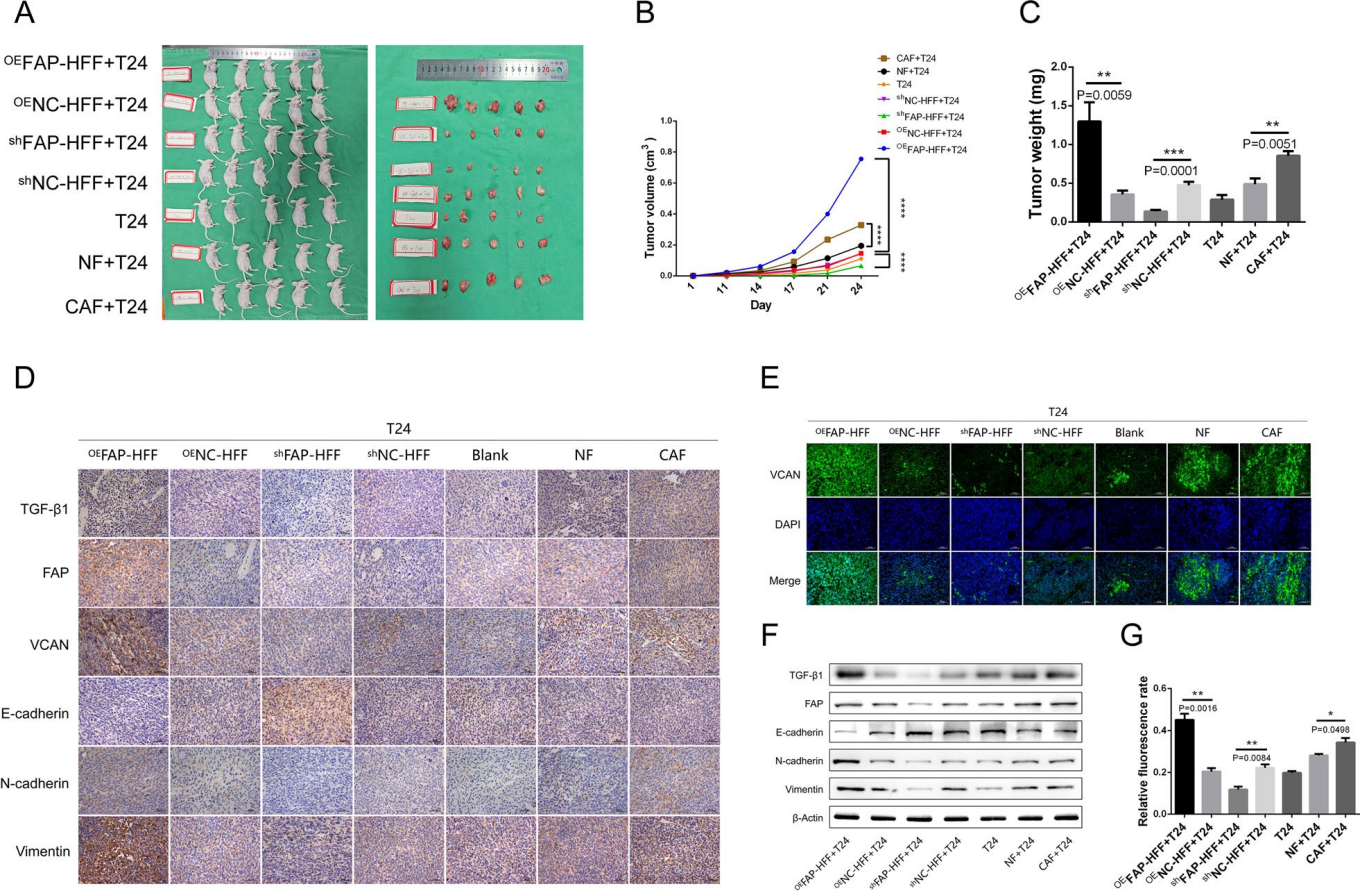
TGF-β1/FAP/VCAN axis promotes stromal fibroblast-mediated EMT in bladder cancer in vivo. **A**–**C** Subcutaneous xenograft models were established by co-injecting T24 cells with fibroblasts, which demonstrated that stromal fibroblasts facilitate tumor formation and growth in vivo. CAF has a stronger tumor-promoting effect than NF. Overexpression of FAP enhanced the tumor-promoting effects of stromal fibroblasts, while knockdown of FAP weakened those effects. Immunohistochemistry (**D**), immunofluorescence (**E**, **G**), and WB (**F**) showed that TGF-β1/FAP/VCAN axis regulates bladder cancer EMT in vivo. CAF has a stronger EMT-inducing effect than NF. Overexpression of FAP enhanced the EMT-inducing effects of stromal fibroblasts, while knockdown of FAP weakened those effects

In order to verify the function of TGF-β1/FAP/VCAN axis in vivo, subcutaneous xenograft models were established by co-injecting T24 cells with fibroblasts (Fig. 8A). We found that stromal fibroblasts facilitate tumor formation and growth in vivo (Fig. 8A–C). CAFs has a stronger tumor-promoting and EMT-inducing effect than NFs. Overexpression of FAP enhanced the tumor-promoting and EMT-inducing effects of stromal fibroblasts, while knockdown of FAP weakened those effects (Fig. 8C–G, Additional file 9A–F). In addition, we demonstrated that the TGF-β1/FAP/VCAN axis regulates bladder cancer EMT in subcutaneous xenograft models (Fig. 8D–G; Additional file 9A–F). Our in vivo results were consistent with the above cytological results.

### FAP, VCAN, N-cadherin, and Vimentin are potential biomarkers for BLCA

**Fig. 9:**
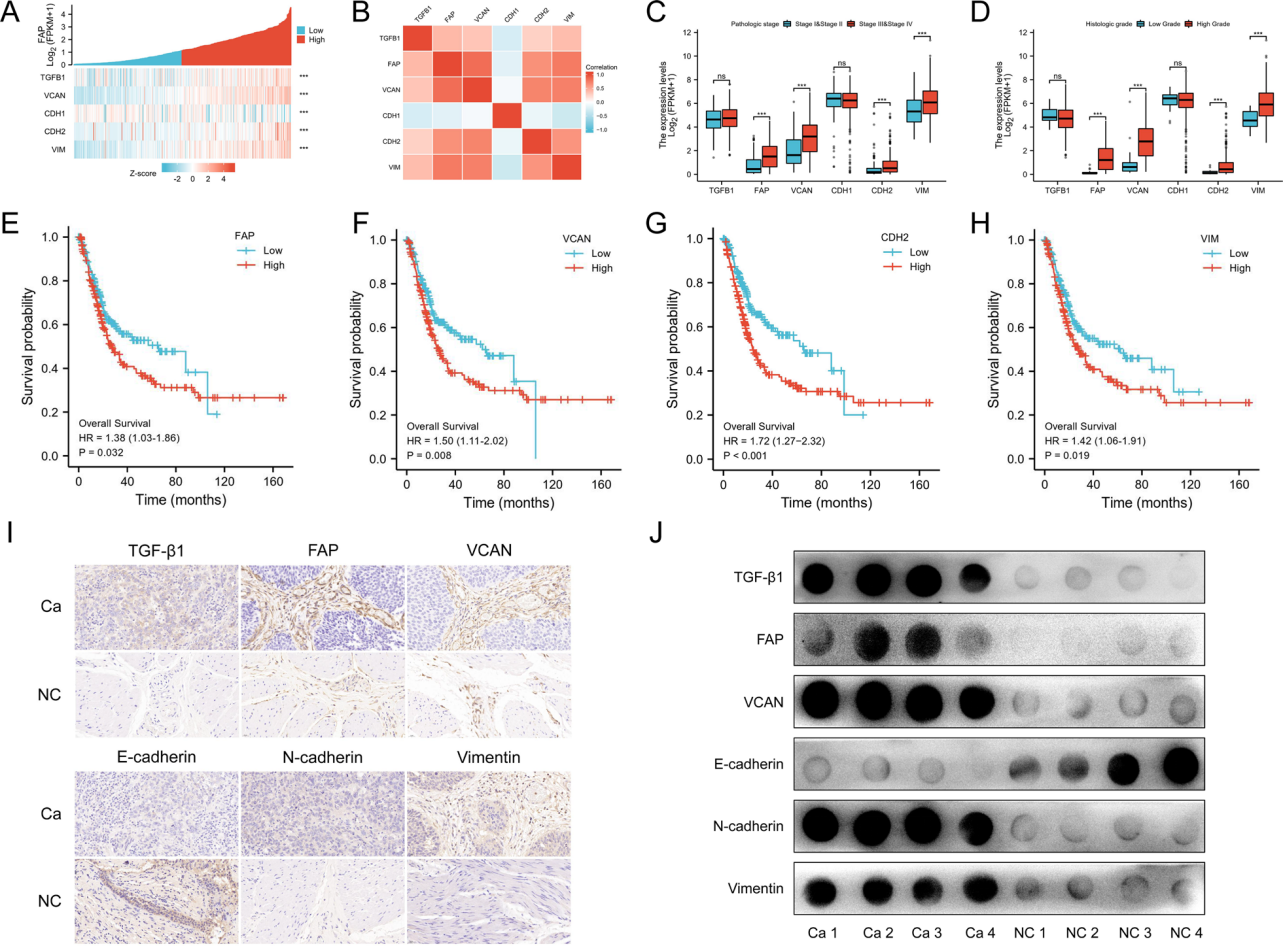
Clinical relevance of TGF-β1, FAP, VCAN, E-cadherin, N-cadherin, and Vimentin in BLCA. **A** According to a heat map analysis of FAP single gene co-expression, FAP is positively co-expressed with TGF-β1, VCAN, N-cadherin, and Vimentin and negatively co-expressed with E-cadherin. **B** The heat map of molecular correlation shows that TGF-β1, FAP, VCAN, N-cadherin, and Vimentin are positively correlated with each other, while E-cadherin is negatively correlated with each. **C** The expression levels of FAP, VCAN, N-cadherin, and Vimentin in stage III–IV of bladder cancer are significantly higher than those in stage I–II. **D** FAP, VCAN, N-cadherin, and Vimentin are significantly higher in high-grade BLCA than in low-grade. The survival curve showed that OS is significantly reduced in BLCA patients with a high expression of (**E**) FAP, (**F**) VCAN, (**G**) N-cadherin, and (**H**) Vimentin. Immunohistochemistry (**I**) and dot blot (**J**) shows that the expression of TGF-β1, FAP, VCAN, N-cadherin, and Vimentin in BLCA tissues is significantly higher than in normal tissues, while the expression of E-cadherin in BLCA tissues is lower than in normal tissues

Bioinformatics analysis was used to validate the expression of TGF-β1, FAP, VCAN, E-cadherin, N-cadherin, and Vimentin at the histological level and their clinical relevance. Firstly, we analyzed the correlation between TGF-β1, FAP, VCAN, E-cadherin, N-cadherin, and Vimentin by means of a FAP single gene co-expression heat map (Fig. 9A) and molecular correlation heat map (Fig. 9B). The analysis results were consistent with our cytological results; a positive correlation was observed between TGF-β1, FAP, VCAN, N-cadherin, and Vimentin, while a negative correlation was observed between E-cadherin and all the above (Fig. 9A, B).

An analysis of clinical correlations showed that FAP, VCAN, N-cadherin, and Vimentin expression levels were significantly higher in stage III-IV BLCA compared to stage I–II (Fig. 9C), and that high-grade BLCA exhibited greater expression than low-grade (Fig. 9D). BLCA with lymphovascular invasion expressed more FAP and VCAN than without lymphovascular invasion (Additional file 10A). In overall survival (OS) and disease-specific survival (DSS) (Additional file 10B, C) events, FAP, VCAN, N-cadherin, and Vimentin expression in the dead group was higher than that in the alive group. In progression-free interval (PFI) events, VCAN and N-cadherin were more highly expressed in the dead group (Additional file 10D). The survival curve showed that OS was significantly reduced in BLCA patients with high expression of FAP, VCAN, N-cadherin, and Vimentin (Fig. 9E–H). Immunohistochemistry (Fig. 9I; Additional file 10E) and dot blot (Fig. 9I; Additional file 10F) showed that the expression of TGF-β1, FAP, VCAN, N-cadherin, and Vimentin in BLCA tissues was significantly higher than in normal tissues, while the expression of E-cadherin in BLCA tissues was lower than in normal tissues. These results suggest that FAP, VCAN, N-cadherin, and Vimentin are associated with the pathology, histology, prognosis, and survival of BLCA patients. Furthermore, FAP, VCAN, N-cadherin, and Vimentin are potential biomarkers for BLCA. Therefore, TGF-β1 derived from stromal fibroblasts is a critical mediator of crosstalk between stromal fibroblasts and BLCA cells, which dominates stromal fibroblast-mediated EMT of BLCA cells via the FAP/VCAN axis to promote the invasion and metastasis of BLCA (Additional file 10G).

## Discussion

BLCA is a complex pathological process with multiple steps and stages caused by various carcinogenic factors. Cancer progression is characterized by a high recurrence rate and invasion risk, as well as multicentric lesions. From a macro perspective, its pathogenesis is affected by both internal genetic factors and external environmental factors. Microscopically, its pathogenesis was influenced not only by abnormal changes in bladder urothelial cells, but also by crosstalk with stromal cells, immune cells, cytokines, ECM, and inflammatory mediators. Although patients with muscle-invasive BLCA can remove local lesions through radical cystectomy and urinary diversion, they face many challenges, including surgical complications, recurrence, metastasis, and poor survival after surgery [15, 16]. Therefore, elucidating the mechanism of invasion and metastasis may help to identify potential risk factors with pro-invasion and pro-metastasis effects. This will aid in clinicians taking appropriate measures to prevent BLCA progression to advanced stages with local invasion or even distant metastasis, which will be of tremendous significance in improving the prognosis of patients.

EMT is one of the essential molecular regulatory mechanisms for BLCA invasion and metastasis. In response to various stimuli, epithelial cells gradually lose their epithelial phenotype (E-cadherin, Catenin) and acquire an interstitial phenotype (N-cadherin, Vimentin), ultimately increasing their ability to migrate and invade [17]. Abnormalities in epigenetic enzymes, protein-coding, and non-coding genes can regulate the EMT of BLCA cells [18]. Many studies have focused on EMT induced by tumor cells themselves. With further study, researchers found that the TME also has an imperative regulatory role in EMT. During the occurrence and development of tumors, NFs in local tissues are activated into CAFs. These cells become one of the predominant cell types in the TME and act as abettors of tumor progression at the crossroads of EMT [19]. For example, Xu et al. found that the CAF-derived cytokine CCL5 can activate the HIF1α/ZEB1 axis, which results in EMT and lung metastasis of hepatocellular carcinoma, indicating that activation of the CAF-mediated EMT pathway may lead to poor patient outcomes [20].

CAF-mediated EMT is a hot topic in current research. However, due to the complexity of the TME, heterogeneity of CAFs, and instability of primary cells, increasing our understanding of CAF-mediated EMT remains difficult. CAFs mediate tumor progression and therapeutic resistance by inducing EMT, activating survival signaling pathways and stem-related procedures, as well as metabolic reprogramming; this highlights the possibility of explicitly eliminating the progression of some tumor subtypes by targeting CAFs with tumor-promoting effects [21]. Researchers successfully isolated and identified two myofibroblast subgroups, CAF-S1 and CAF-S4, from metastatic lymph nodes of breast cancer and found that the CAF-S1 subgroup stimulated breast cancer cell migration and EMT through the CXCL-12 and TGF-β pathway, while the CAF-S4 subgroup induced breast cancer cell invasion through the NOTCH signaling pathway [22]. To reveal the molecular mechanism of CAF-mediated EMT in BLCA invasion and metastasis, we successfully isolated primary CAFs and NFs from freshly isolated bladder tissues. Notably, stromal fibroblasts in bladder cancer tissues include inactive NFs and activated CAFs, and may even be in an intermediate state between them. Therefore, it is not yet clear how CAFs differ from NFs. The commonly used method for CAFs identification reported in the literature was to detect the expression of activation markers such as FAP, FSP1, α-SMA, and CD90 in stromal fibroblasts. The above markers were detected by qRT-PCR, WB, and immunofluorescence staining, all of which confirmed that CAFs were isolated from cancer tissues rather than NFs (Fig. 1).

The TGF-β signaling pathway plays multiple roles in the physiopathological processes of our bodies, including embryonic development, organ formation, tissue repair, homeostasis, tumor invasion, metastasis, immune escape, and therapeutic resistance [23, 24]. Activated by carcinogenic factors, TGF-β signaling can regulate tumor initiation, distant metastasis and colonization, immune escape, and therapeutic resistance by inducing tumor cell EMT, activating NFs into CAFs, ECM reconstruction, and so on [21, 25]. TGF-β1 was the highest paracrine cytokine secreted by CAFs in breast cancer, which is not only a critical mediator to regulate the crosstalk between stromal cells and tumor cells, but also a key abettor for CAF-induced EMT [26]. In ovarian cancer, CAFs secrete a large amount of the ECM protein, Periodin (POSTEN), to promote EMT, migration, and invasion of ovarian cancer cells by activating the PI3K/AKT signaling pathway under the stimulation of TGF-β1. It was suggested that TGF-β1-induced upregulation of POSTEN in CAFs was indispensable for matrix fibrosis and the pro-metastatic microenvironment [27]. Similarly, the role of TGF-β signaling in EMT was also demonstrated in colorectal cancer; CAFs promote EMT and invasion of colorectal cancer cells by activating the TGF-β/SMAD axis and attenuating the expression of FLRT3 [28].

Our study discovered that CM derived from stromal fibroblasts (not only CAF-CM but also NF-CM) had a pro-migration effect on BLCA cells. Given the significance of TGF-β1 signaling in EMT, it is imperative that we verify the expression of TGF-β1 in BLCA at the histological level. By analyzing TCGA data of BLCA, we verified that the expression of TGF-β1 was significantly higher than TGF-β2 and TGF-β3 in BLCA tissues. The same results were obtained in the ELISA assay, showing that primary stromal fibroblasts secreted significantly more TGF-β1 than TGF-β2 and TGF-β3. Therefore, TGF-β1 was selected for further study. We noted that the expression and extracellular secretion of TGF-β1 in CAFs and NFs were considerably higher than in T24 cells. Although there was no significant difference in the extracellular secretion of NFs and CAFs by ELISA, the focus of our study was on the tumor-promoting effect of TGF-β1 which derived from stromal fibroblasts, rather than the difference in the secretion of TGF-β1 by CAF-CM and NF-CM. NFs and CAFs are both stromal fibroblasts, representing two different stromal fibroblast states. In addition, CAFs had the highest expression level of TGF-β1 among the three cell groups. Therefore, the primary molecule responsible for BLCA cell migration is thought to be TGF-β1 derived from stromal fibroblasts, especially from CAFs (Fig. 2).

To further clarify the effect of TGF-β1 derived from stromal fibroblasts, we set up a co-culture system and assessed the pro-migration and pro-invasion effects of CAF-CM and NF-CM on BLCA cells using a wound healing assay and transwell assay, respectively. Subsequently, we confirmed the pro-EMT effect of CAF-CM and NF-CM by detecting the expression of EMT markers in T24 cells from the co-culture system. CAF-CM provided a significantly higher tumor-promoting effect than NF-CM, which was due to CAFs representing an activation state of NFs. In line with this, CAFs secreted more tumor-promoting factors (not only TGF-β1) than NFs, and had a more powerful tumor-promoting effect. Furthermore, to demonstrate the tumor-promoting effect of TGF-β1 among numerous tumor-promoting factors, we set up a reverse control group by TGF-β1 neutralizing antibody, which proved that stromal fibroblast-derived TGF-β1 promotes BLCA cell migration, invasion, and EMT. Conversely, antagonizing TGF-β1 could attenuate the stromal fibroblast-mediated EMT effect (Fig. 3). In addition, we found that there were notable differences between the experimental group and the control IgG group in some figures. To address this controversy, we explain that IgG control may affect not only the microenvironment, but also the phenotype and function of tumor cells and fibroblasts in co-culture systems. It is critical to note that we had the right overall trend in the following experiments and the conclusion was not affected by control IgG.

As a research hot spot in recent years and one of the most common molecular markers of CAFs, FAP is a glycoprotein expressed primarily on the cell surface of reactive stromal fibroblasts of epithelial tumors and granulation tissue during wound healing [29]. Recently, FAP has been used for CAF identification and phenotype research, and its involvement in CAF-mediated EMT has been demonstrated in some tumors [30]. For instance, exosomes derived from BLCA cells can induce normal bladder fibroblast activation and endow them with CAF characteristics, including overexpression of FAP and α-SMA. Mechanically, CAF-induced EMT in BLCA cells relies on paracrine IL-6. Clinically, IL-6 is highly expressed in muscle-invasive BLCA tissue, which is significantly correlated with the expression of the CAF marker α-SMA [31]. Similarly, TGF-β1 can trigger the activation of CAFs in breast cancer; activated CAFs promote tumor invasion, metastasis, and EMT by inducing autophagy and overexpressing FAP. According to these data, CAF-mediated EMT through autophagy and overexpression of FAP is critical for invasion and metastasis of breast cancer [32].

We next aimed to identify the relationship between TGF-β1 and FAP, and how these two molecules regulate stromal fibroblast-mediated function in BLCA. We found that the expression of FAP was upregulated in both BLCA tissues and primary CAFs based on bioinformatics analyses, qRT-PCRs, and WBs. Furthermore, exogenous TGF-β1 induced primary NF and fibroblast cell line HFF, showing that TGF-β1 promotes stromal fibroblasts into an activated state with CAF-like features by upregulating FAP (Fig. 4). Moreover, we confirmed the association between the TGF-β1/FAP axis and EMT of BLCA cells. T24 cells were found to migrate faster when co-cultured with ^OE^FAP-HFF-CM. Interestingly, HFF-CM may promote migration and invasion of T24 cells in wound healing and transwell assays; however, the migration and invasion effects were dramatically amplified after using exogenous TGF-β1 to induce HFF. The TGF-β1 neutralizing antibody significantly reduced the amplification effect induced by TGF-β1. Moreover, HFF-CM (stimulated or not stimulated) promoted EMT of T24 cells in different co-culture systems. As expected, the pro-EMT effects of HFF-CM were significantly amplified after TGF-β1 induction or overexpression FAP, whereas blocking TGF-β1 with a neutralizing antibody could reverse the amplification effect induced by TGF-β1. These results indicate that TGF-β1 promotes stromal fibroblast-mediated EMT in BLCA cells by upregulating FAP (Fig. 5; Additional file 6).

To determine whether downstream factors of FAP affect EMT in BLCA cells, we performed GSEA enrichment analysis, which revealed a positive correlation between FAP differential protein-coding genes and EMT in BLCA. A total of 138 differentially expressed proteins were identified by the human cytokine antibody array, VCAN being the most significant among them (Fig. 6). VCAN is a macromolecular chondroitin sulfate proteoglycan, which is one of the main protein components of the ECM. It participates in regulating cell proliferation, migration, adhesion, and ECM remodeling [33]. As an essential mediator of cell-to-cell and cell-to-ECM communication, the role of VCAN secreted by CAF has gained increasing attention. However, the exact regulatory mechanisms are not yet fully elucidated, especially in regulating the phenotype of BLCA. Several studies have shown that CAFs secrete VCAN to promote the malignant phenotype of gastrointestinal tumors; high expression of VCAN is a high-risk factor for gastrointestinal tumor treatment resistance and poor prognosis [34, 35].

Based on TCGA data, we found that BLCA tissues expressed significantly more VCAN than adjacent normal tissues. Results from the molecular correlation showed a positive correlation between FAP and VCAN. A series of cytological assays revealed that VCAN is a downstream molecule of FAP. Its expression and paracrine activity were dramatically upregulated in stromal fibroblasts expressing high levels of FAP (Fig. 6; Additional file 7).

It has also been reported that VCAN can enhance the migration and invasion of breast cancer cells by promoting Snail-mediated EMT. The expression of VCAN and Snail in breast cancer tissue is significantly positively correlated [36]. Additionally, researchers discovered the role of VCAN in the tumor immune microenvironment. In tissues with high expression of VCAN, they observed enhanced proliferation and migration of cells, an increased number of endothelial cells, myofibroblasts, M1 macrophages, and other inflammatory cells, as well as increased expression of Ki67, CD31, α-SMA, and Periostin; TGF-β signal activation; and Smad2/3 signal upregulation [37]. Likewise, another study revealed that VCAN regulates the abundance and activation of dendritic cells to induce the formation of an inflammatory TME by mediating matrix remodeling [38].

These collective findings support VCAN regulating numerous biological processes in cancers through diverse molecular mechanisms; however, the regulatory mechanisms require further investigation. The purpose of this study was to explore the communication between TGF-β1/FAP/VCAN axis in fibroblasts and EMT in BLCA cells. As expected, we confirmed a consistent expression trend between VCAN and FAP under TGF-β1 induction. The expression of VCAN in NFs and HFF was upregulated by TGF-β1 induction, and the TGF-β1 neutralizing antibody attenuated the upregulation of VCAN. ^OE^VCAN-HFF-CM promoted migration and invasion of T24 cells in wound healing assays and transwell assays. Consistently, ^OE^VCAN-HFF-CM showed similar pro-EMT effects as primary CAFs and ^OE^FAP-HFFs (Fig. 7; Additional file 8). Mechanically, the PI3K/AKT pathway was significantly enriched in the GO/KEGG analysis (Fig. 6B). In line with this, we observed major increases in PI3K and AKT1 mRNA levels in T24 cells in response to ^OE^VCAN-HFF-CM (Fig. 7J). Our research indicates that the TGF-β1/FAP axis promotes stromal fibroblast-mediated EMT in BLCA cells by upregulating VCAN, and PI3K/AKT signaling may be responsible for VCAN's role.

Above all, we revealed that the TGF-β1/FAP/VCAN axis promotes stromal fibroblast-mediated EMT in bladder cancer in cytological experiments. As a further demonstration of the reliability of our findings, we created subcutaneous xenograft models and demonstrated that the TGF-β1/FAP/VCAN axis regulates bladder cancer EMT in vivo (Fig. 8; Additional file 9). Both our in vitro and in vivo results were consistent.

Lastly, we verified the potential clinical value of TGF-β1, FAP, VCAN, E-cadherin, N-cadherin, and Vimentin using pathological, histological, and prognostic survival analyses (Fig. 9; Additional file 10). Strikingly, our cytological results were consistent with the molecular correlation heat map and FAP single gene co-expression heat map. The correlation between TGF-β1, FAP, VCAN, N-cadherin, and Vimentin was positive, while the correlation between E-cadherin and all the above was negative. Clinically, FAP, VCAN, N-cadherin, and Vimentin were associated with pathological stage, histological grade, OS event, and DSS event of BLCA. FAP and VCAN were associated with lymphovascular invasion. VCAN and N-cadherin were associated with PFI events. Immunohistochemistry and dot blot confirmed that BLCA tissues expressed higher TGF-β1, FAP, VCAN, N-cadherin, and Vimentin, and lower E-cadherin than adjacent normal tissues. OS was significantly reduced in BLCA patients with high expression of FAP, VCAN, N-cadherin, and Vimentin. These results indicate that FAP, VCAN, N-cadherin, and Vimentin are associated with pathology, histology, prognosis, and survival of BLCA. Furthermore, FAP, VCAN, N-cadherin, and Vimentin are potential biomarkers for BLCA diagnosis and treatment.

## Conclusions

Our research provides strong evidence that TGF-β1 in the TME is mainly derived from stromal fibroblasts, especially from CAFs. TGF-β1 is a key mediator that regulates the interaction between stromal fibroblasts and BLCA cells. On the one hand, TGF-β1 could induce the activation of normal fibroblasts and endow them with CAF-like characteristics and phenotypes. On the other hand, activated stromal fibroblasts or CAFs secrete more TGF-β1 to the TME, forming a dynamic, positive feedback loop in the TME and jointly building a tumor-promoting microenvironment under the command of TGF-β1. Most importantly, our study has also revealed a new mechanism whereby TGF-β1 dominates stromal fibroblast-mediated EMT of BLCA cells by the FAP/VCAN axis (Additional file 10G), and identified potential biomarkers of BLCA: FAP, VCAN, N-cadherin, and Vimentin. In addition, VCAN may also function through the PI3K/AKT signaling pathway, but more research is needed. These results not only enhance our understanding of BLCA invasion and metastasis, but also provide potential strategies for diagnosis, treatment, and prognosis. However, there are some defects in our study that need to be further improved. These defects include the use of only one BLCA cell line, and insufficient research on VCAN's downstream signaling pathway. Lastly, although a wealth of pre-clinical data was obtained, much effort is still needed to translate CAF-directed or TME-directed anti-cancer strategies from the bench to the clinic due to the complexity of the tumor microenvironment and tumor organism environment.

## Supplementary Information

**Additional file 1:** Primer sequences of quantitative real-time.

**Additional file 2:** Antibodies information of Western blotting.

**Additional file 3:**ELISA kits information.

**Additional file 4:**Antibodies information of Immunofluorescence.

**Additional file 5:**Antibodies information of Immunochemistry.

**Additional file 6:**(A) qRT-PCR verifies that the mRNA expression of FAP in ^OE^FAP-HFFs is significantly higher than in ^NC^FAP-HFFs and T24 cells. (B, C) WB indicates that the protein expression of FAP in ^OE^FAP-HFFs is significantly higher than in ^NC^FAP-HFFs and T24 cells. (D, E) WB show that HFF-CM (stimulated or not stimulated) has a pro-EMT effect, with downregulated E-cadherin and upregulated N-cadherin and Vimentin. The pro-EMT effects of HFF-CM were significantly amplified after TGF-β1 induction or overexpression FAP, while the TGF-β1 neutralizing antibody reverses the pro-EMT effects of TGF-β1 induction.

**Additional file 7:** (A) VCAN expression is significantly higher in BLCA tissues than in adjacent normal tissues. (B) Immunofluorescence shows that the fluorescence intensity of VCAN in CAFs is notably brighter than in NFs, and in ^OE^FAP-HFFs it is notably brighter than in ^NC^FAP-HFFs.

**Additional file 8:** (A) Immunofluorescence shows that the fluorescence intensity of VCAN in HFFs is markedly enhanced under the induction of TGF-β1; TGF-β1 neutralizing antibody significantly reduces this enhanced fluorescence intensity. (B) qRT-PCR verifies that the expression of VCAN in ^OE^VCAN-HFFs is significantly higher than in ^NC^VCAN-HFFs and T24 cells. (C) Immunofluorescence reveals that the VCAN fluorescence intensity in ^OE^VCAN-HFFs is notably brighter than in ^NC^VCAN-HFFs and T24 cells. (D) ELISA shows that the concentration of VCAN in the ^OE^VCAN-HFF supernatant is significantly higher than in ^NC^VCAN-HFF and T24 cell supernatant. (E) WB show that ^OE^VCAN-HFF-CM can downregulate E-cadherin as well as upregulate N-cadherin and Vimentin.

**Additional file 9:** qRT-PCR (A, B), WB(C, D), and immunohistochemistry (E, F) showed that TGF-β1/FAP/VCAN axis regulates bladder cancer EMT in vivo. CAF has a stronger EMT-inducing effect than NF. Overexpression of FAP enhanced the EMT-inducing effects of stromal fibroblasts, while knockdown of FAP weakened those effects.

**Additional file 10:** (A) BLCA with lymphovascular invasion expressed more FAP and VCAN than without lymphovascular invasion. In OS (B) and DSS (C) events, FAP, VCAN, N-cadherin, and Vimentin expression in the deceased group is higher than in the alive group. (D) In PFI events, VCAN and N-cadherin are more highly expressed in the deceased group. Immunohistochemistry (E) and dot blot (F) shows that the expression of TGF-β1, FAP, VCAN, N-cadherin, and Vimentin in BLCA tissues is significantly higher than in adjacent normal tissues, while the expression of E-cadherin in BLCA tissues is lower than in adjacent normal tissues. (G) TGF-β1 dominates stromal fibroblast-mediated EMT of bladder cancer cells via the FAP/VCAN axis to promote the invasion and metastasis of BLCA.

## Data Availability

Not applicable.

## Abbreviations

α-SMA: α-Smooth muscle actin
BLCA: Bladder cancer
CAF: Cancer-associated fibroblasts
CM: Conditioned medium
DSS: Disease-specific survival
ECM: Extracellular matrix
ELISA: Enzyme-linked immunosorbent assay
EMT: Epithelial–mesenchymal transition
FAP: Fibroblast activation protein
FBS: Fetal bovine serum
FSP1: Fibroblast-specific protein 1
GSEA: Gene set enrichment analysis
GO/KEGG: Gene Ontology and Kyto Encyclopedia of Genes and Genomes
NF: Normal fibroblasts
OS: Overall survival
PFI: Progression-free interval
qRT-PCR: Quantitative real-time PCR
TGF-β: Transforming growth factor β
TME: Tumor microenvironment
VCAN: Versican
WB: Western blotting
