# Supplementary figures and images for: TGF-β1 dominates stromal fibroblast-mediated EMT via the FAP/VCAN axis in bladder cancer cells
Source: J Transl Med. 2023 Jul 17;21:475. doi: 10.1186/s12967-023-04303-3 (PMC10351189; doi:10.1186/s12967-023-04303-3)

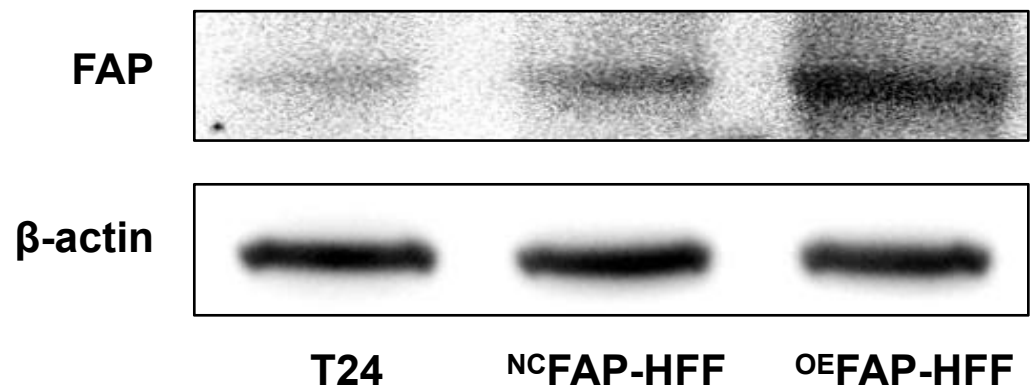

Supplement: Supplementary file 6 — Additional file 6:(A) qRT-PCR verifies that the mRNA expression of FAP in OEFAP-HFFs is significantly higher than in NCFAP-HFFs and T24 cells. (B, C) WB indicates that the protein expression of FAP in OEFAP-HFFs is significantly higher than in NCFAP-HFFs and T24 cells. (D, E) WB show that HFF-CM (stimulated or not stimulated) has a pro-EMT effect, with downregulated E-cadherin and upregulated N-cadherin and Vimentin. The pro-EMT effects of HFF-CM were significantly amplified after TGF-β1 induction or overexpression FAP, while the TGF-β1 neutralizing antibody reverses the pro-EMT effects of TGF-β1 induction. [file 12967_2023_4303_MOESM6_ESM.zip › Additional file 6B.pdf]

The expression of VCAN  
 $\text{Log}_2(\text{FPKM}+1)$

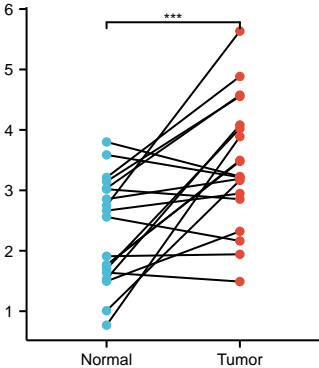

Supplement: Supplementary file 7 — Additional file 7: (A) VCAN expression is significantly higher in BLCA tissues than in adjacent normal tissues. (B) Immunofluorescence shows that the fluorescence intensity of VCAN in CAFs is notably brighter than in NFs, and in OEFAP-HFFs it is notably brighter than in NCFAP-HFFs. [file 12967_2023_4303_MOESM7_ESM.zip › Additional file 7A.pdf]

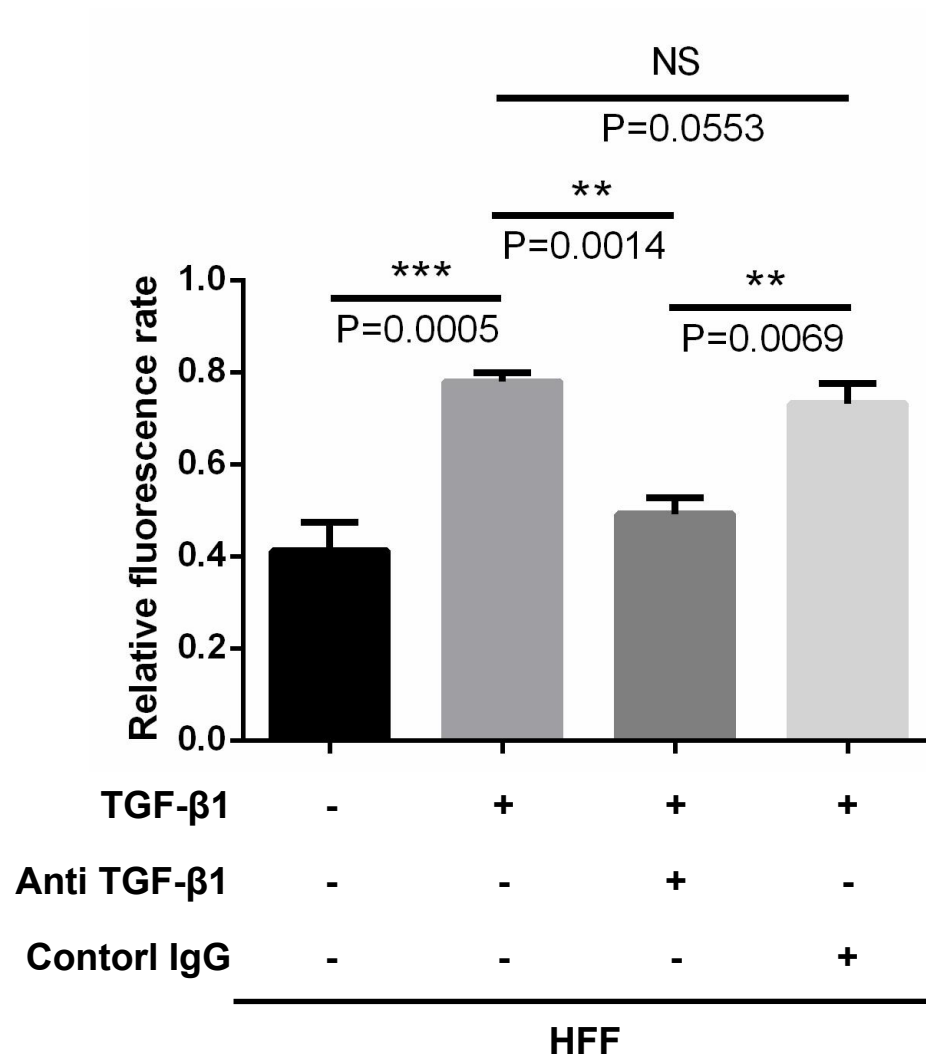

Supplement: Supplementary file 8 — Additional file 8: (A) Immunofluorescence shows that the fluorescence intensity of VCAN in HFFs is markedly enhanced under the induction of TGF-β1; TGF-β1 neutralizing antibody significantly reduces this enhanced fluorescence intensity. (B) qRT-PCR verifies that the expression of VCAN in OEVCAN-HFFs is significantly higher than in NCVCAN-HFFs and T24 cells. (C) Immunofluorescence reveals that the VCAN fluorescence intensity in OEVCAN-HFFs is notably brighter than in NCVCAN-HFFs and T24 cells. (D) ELISA shows that the concentration of VCAN in the OEVCAN-HFF supernatant is significantly higher than in NCVCAN-HFF and T24 cell supernatant. (E) WB show that OEVCAN-HFF-CM can downregulate E-cadherin as well as upregulate N-cadherin and Vimentin. [file 12967_2023_4303_MOESM8_ESM.zip › Additional file 8A.pdf]

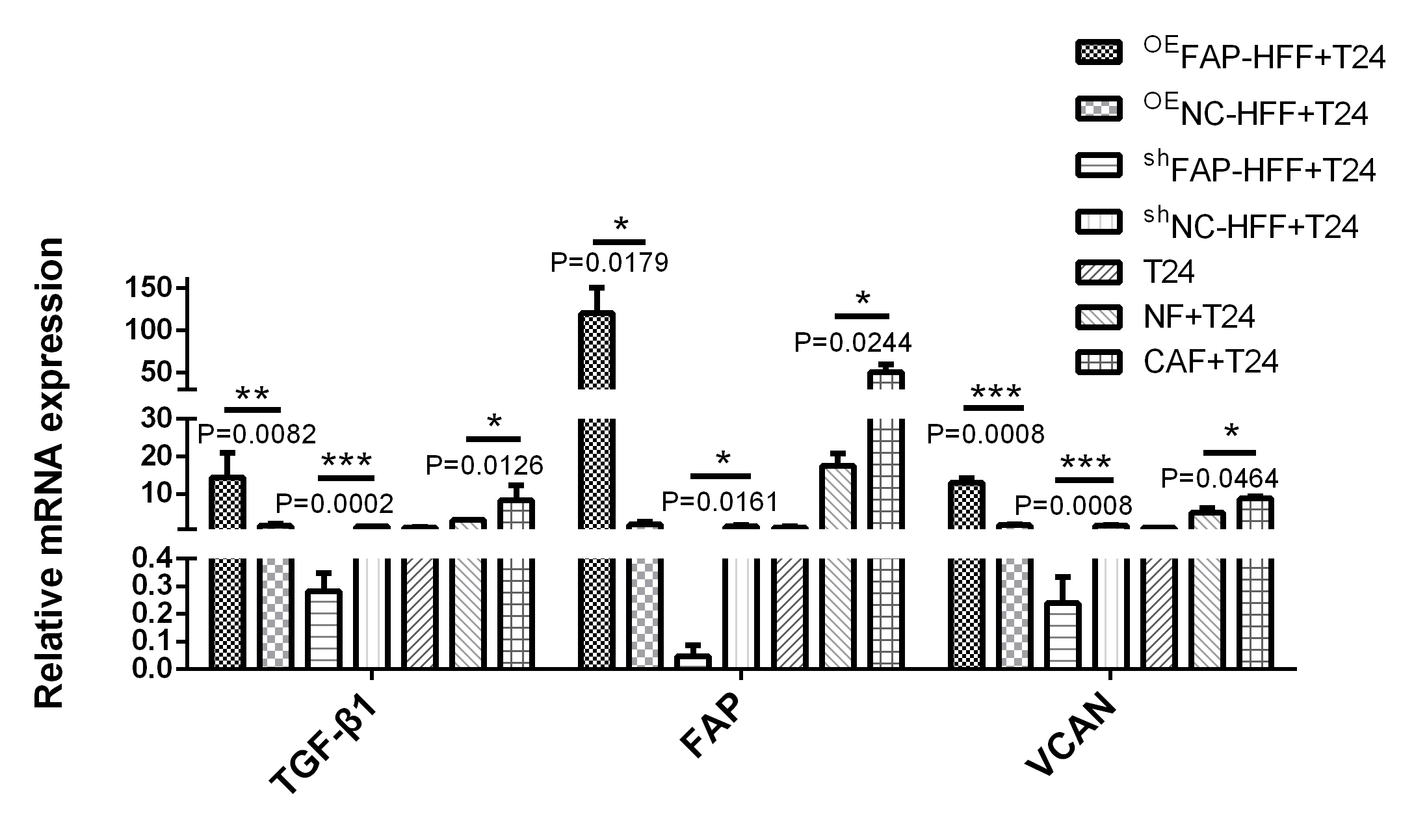

Supplement: Supplementary file 9 — Additional file 9: qRT-PCR (A, B), WB(C, D), and immunohistochemistry (E, F) showed that TGF-β1/FAP/VCAN axis regulates bladder cancer EMT in vivo. CAF has a stronger EMT-inducing effect than NF. Overexpression of FAP enhanced the EMT-inducing effects of stromal fibroblasts, while knockdown of FAP weakened those effects. [file 12967_2023_4303_MOESM9_ESM.zip › Additional file 9A.jpg]

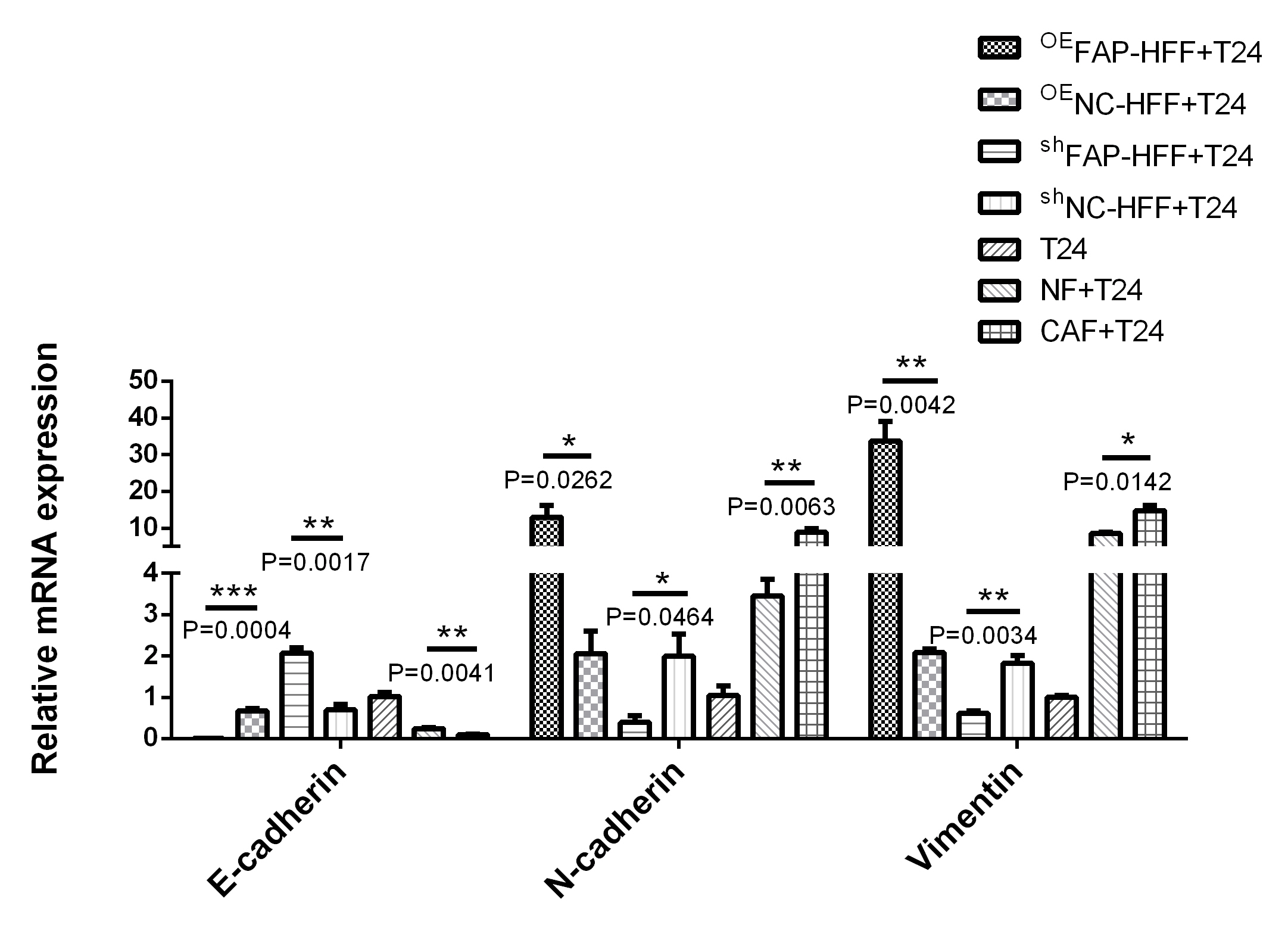

Supplement: Supplementary file 9 — Additional file 9: qRT-PCR (A, B), WB(C, D), and immunohistochemistry (E, F) showed that TGF-β1/FAP/VCAN axis regulates bladder cancer EMT in vivo. CAF has a stronger EMT-inducing effect than NF. Overexpression of FAP enhanced the EMT-inducing effects of stromal fibroblasts, while knockdown of FAP weakened those effects. [file 12967_2023_4303_MOESM9_ESM.zip › Additional file 9B.jpg]

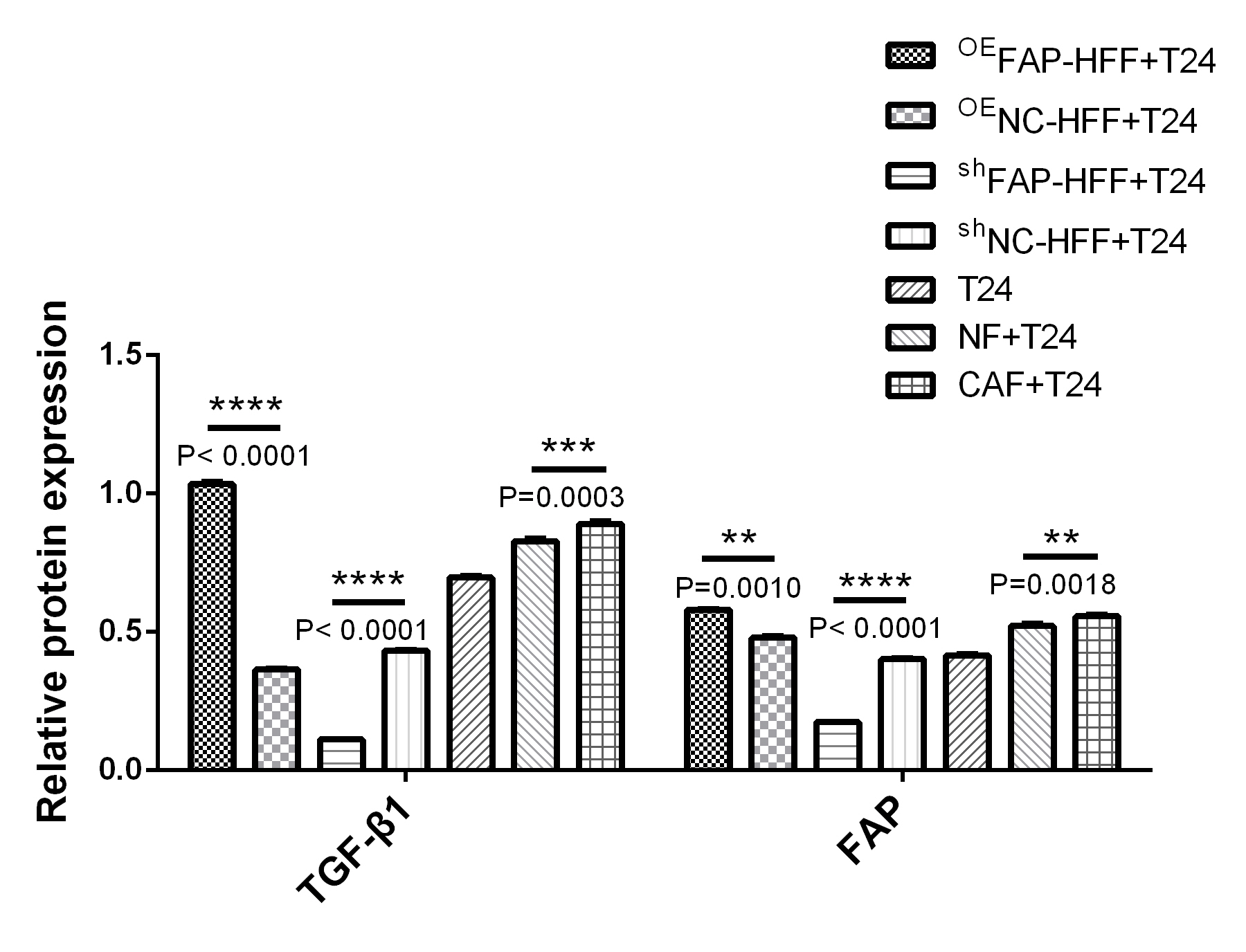

Supplement: Supplementary file 9 — Additional file 9: qRT-PCR (A, B), WB(C, D), and immunohistochemistry (E, F) showed that TGF-β1/FAP/VCAN axis regulates bladder cancer EMT in vivo. CAF has a stronger EMT-inducing effect than NF. Overexpression of FAP enhanced the EMT-inducing effects of stromal fibroblasts, while knockdown of FAP weakened those effects. [file 12967_2023_4303_MOESM9_ESM.zip › Additional file 9C.jpg]

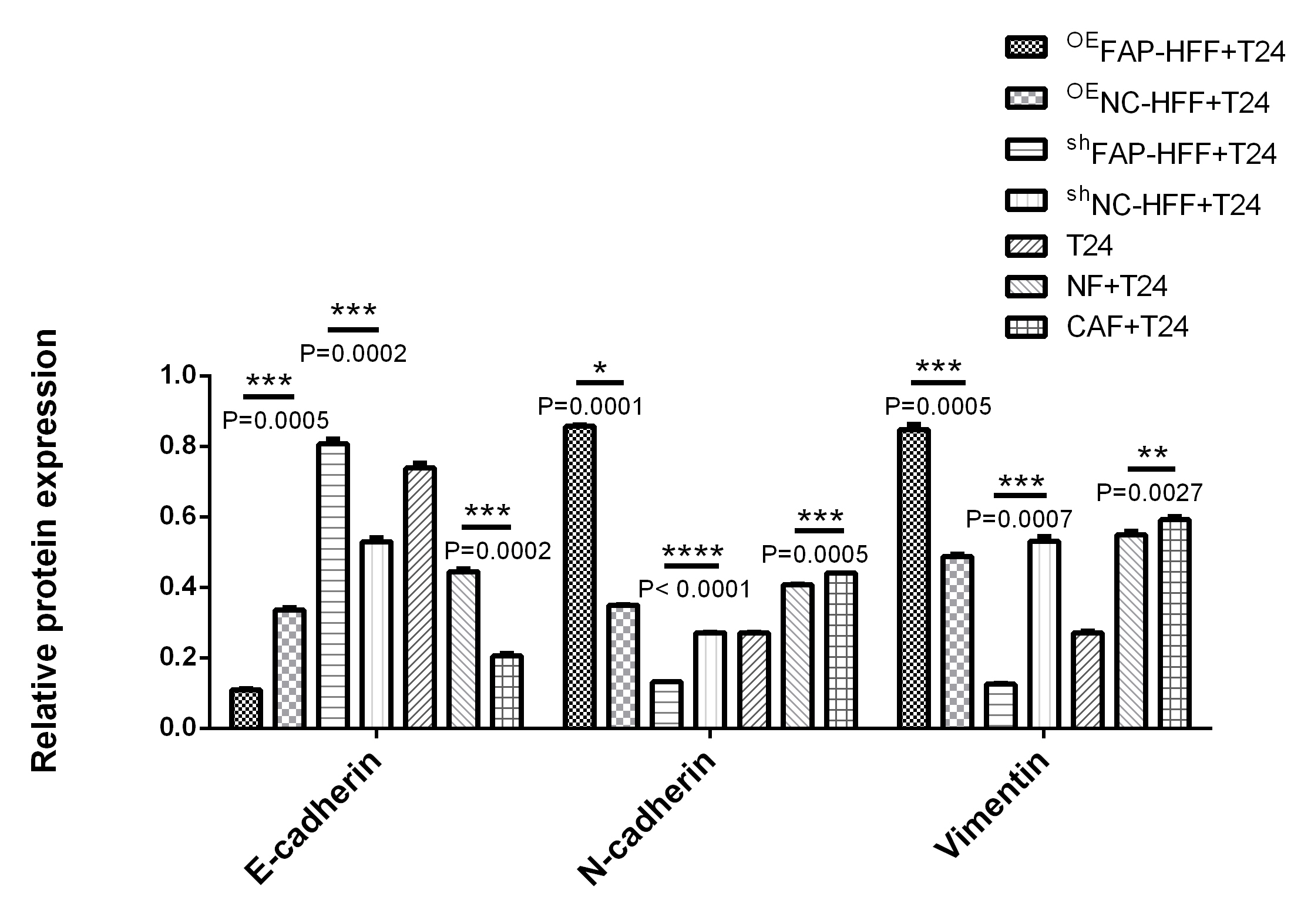

Supplement: Supplementary file 9 — Additional file 9: qRT-PCR (A, B), WB(C, D), and immunohistochemistry (E, F) showed that TGF-β1/FAP/VCAN axis regulates bladder cancer EMT in vivo. CAF has a stronger EMT-inducing effect than NF. Overexpression of FAP enhanced the EMT-inducing effects of stromal fibroblasts, while knockdown of FAP weakened those effects. [file 12967_2023_4303_MOESM9_ESM.zip › Additional file 9D.jpg]

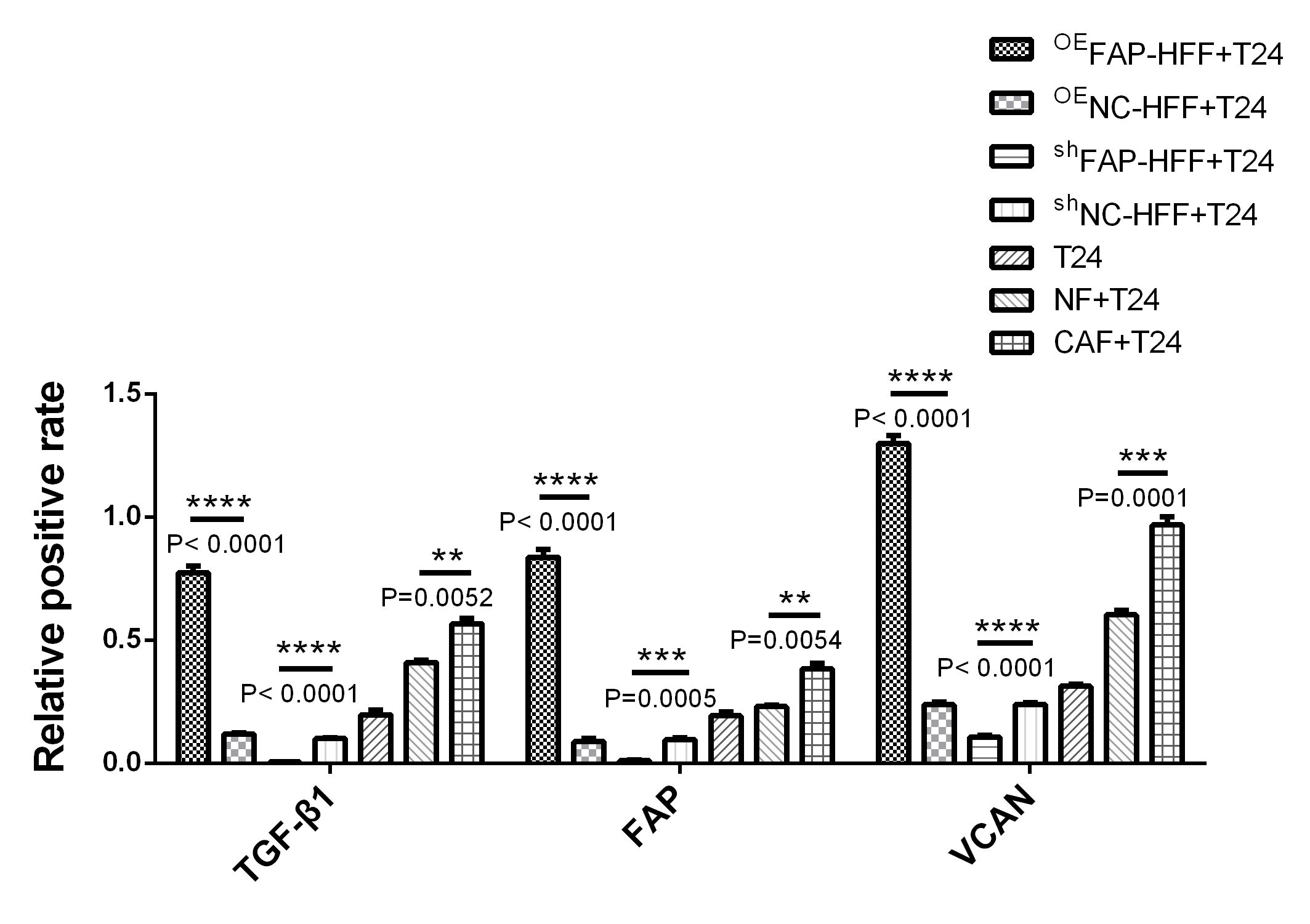

Supplement: Supplementary file 9 — Additional file 9: qRT-PCR (A, B), WB(C, D), and immunohistochemistry (E, F) showed that TGF-β1/FAP/VCAN axis regulates bladder cancer EMT in vivo. CAF has a stronger EMT-inducing effect than NF. Overexpression of FAP enhanced the EMT-inducing effects of stromal fibroblasts, while knockdown of FAP weakened those effects. [file 12967_2023_4303_MOESM9_ESM.zip › Additional file 9E.jpg]

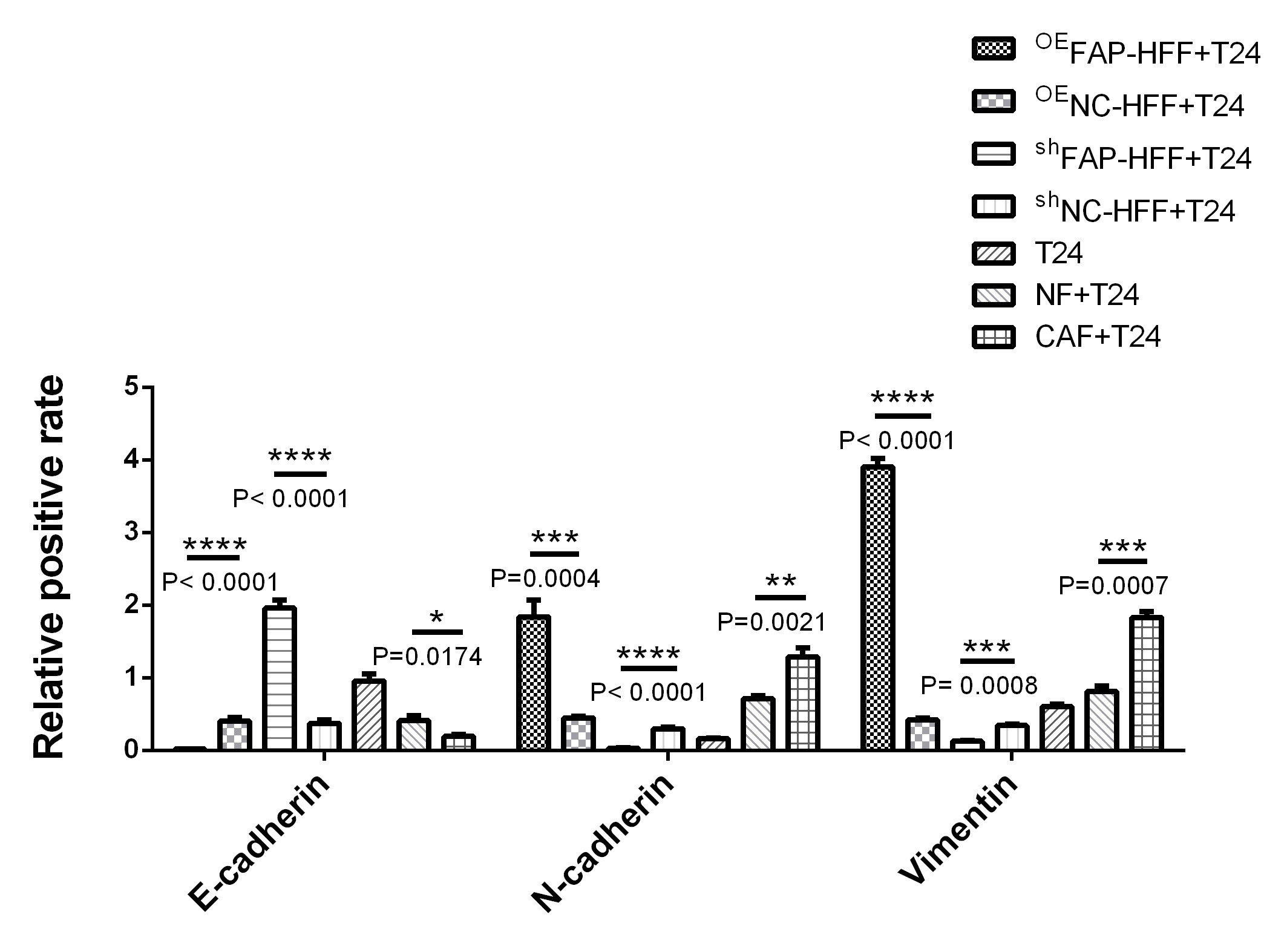

Supplement: Supplementary file 9 — Additional file 9: qRT-PCR (A, B), WB(C, D), and immunohistochemistry (E, F) showed that TGF-β1/FAP/VCAN axis regulates bladder cancer EMT in vivo. CAF has a stronger EMT-inducing effect than NF. Overexpression of FAP enhanced the EMT-inducing effects of stromal fibroblasts, while knockdown of FAP weakened those effects. [file 12967_2023_4303_MOESM9_ESM.zip › Additional file 9F.jpg]

Lymphovascular invasion    No    Yes

The expression levels  
 $\text{Log}_2(\text{FPKM}+1)$

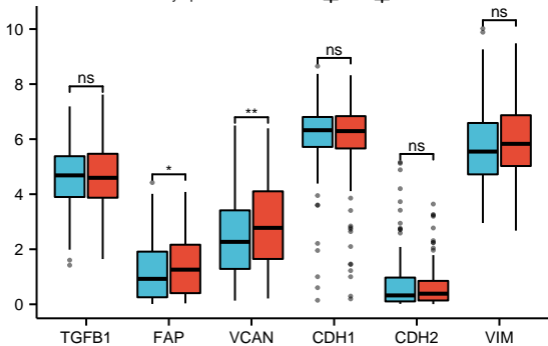

Supplement: Supplementary file 10 — Additional file 10: (A) BLCA with lymphovascular invasion expressed more FAP and VCAN than without lymphovascular invasion. In OS (B) and DSS (C) events, FAP, VCAN, N-cadherin, and Vimentin expression in the deceased group is higher than in the alive group. (D) In PFI events, VCAN and N-cadherin are more highly expressed in the deceased group. Immunohistochemistry (E) and dot blot (F) shows that the expression of TGF-β1, FAP, VCAN, N-cadherin, and Vimentin in BLCA tissues is significantly higher than in adjacent normal tissues, while the expression of E-cadherin in BLCA tissues is lower than in adjacent normal tissues. (G) TGF-β1 dominates stromal fibroblast-mediated EMT of bladder cancer cells via the FAP/VCAN axis to promote the invasion and metastasis of BLCA. [file 12967_2023_4303_MOESM10_ESM.zip › Additional file10A.pdf]

The expression levels  
 $\text{Log}_2(\text{FPKM}+1)$

OS event 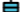 Alive 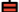 Dead

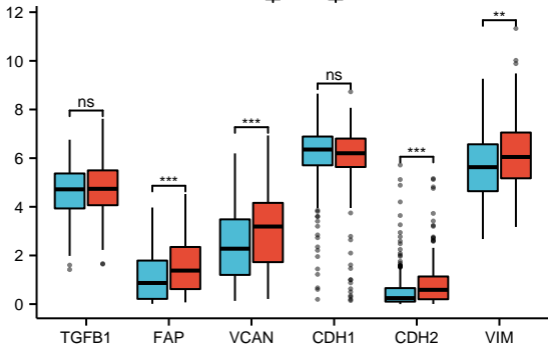

Supplement: Supplementary file 10 — Additional file 10: (A) BLCA with lymphovascular invasion expressed more FAP and VCAN than without lymphovascular invasion. In OS (B) and DSS (C) events, FAP, VCAN, N-cadherin, and Vimentin expression in the deceased group is higher than in the alive group. (D) In PFI events, VCAN and N-cadherin are more highly expressed in the deceased group. Immunohistochemistry (E) and dot blot (F) shows that the expression of TGF-β1, FAP, VCAN, N-cadherin, and Vimentin in BLCA tissues is significantly higher than in adjacent normal tissues, while the expression of E-cadherin in BLCA tissues is lower than in adjacent normal tissues. (G) TGF-β1 dominates stromal fibroblast-mediated EMT of bladder cancer cells via the FAP/VCAN axis to promote the invasion and metastasis of BLCA. [file 12967_2023_4303_MOESM10_ESM.zip › Additional file10B.pdf]

The expression levels  
 $\text{Log}_2(\text{FPKM}+1)$

DSS event 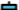 Alive 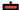 Dead

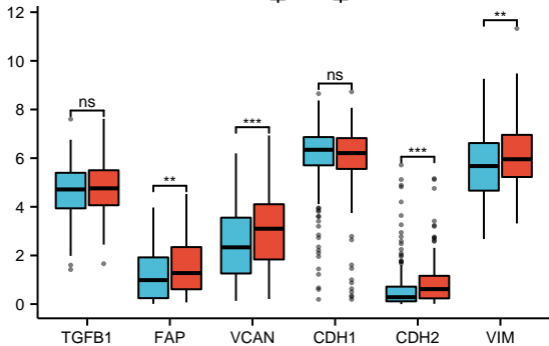

Supplement: Supplementary file 10 — Additional file 10: (A) BLCA with lymphovascular invasion expressed more FAP and VCAN than without lymphovascular invasion. In OS (B) and DSS (C) events, FAP, VCAN, N-cadherin, and Vimentin expression in the deceased group is higher than in the alive group. (D) In PFI events, VCAN and N-cadherin are more highly expressed in the deceased group. Immunohistochemistry (E) and dot blot (F) shows that the expression of TGF-β1, FAP, VCAN, N-cadherin, and Vimentin in BLCA tissues is significantly higher than in adjacent normal tissues, while the expression of E-cadherin in BLCA tissues is lower than in adjacent normal tissues. (G) TGF-β1 dominates stromal fibroblast-mediated EMT of bladder cancer cells via the FAP/VCAN axis to promote the invasion and metastasis of BLCA. [file 12967_2023_4303_MOESM10_ESM.zip › Additional file10C.pdf]

The expression levels  
 $\text{Log}_2(\text{FPKM}+1)$

PFI event 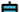 Alive 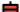 Dead

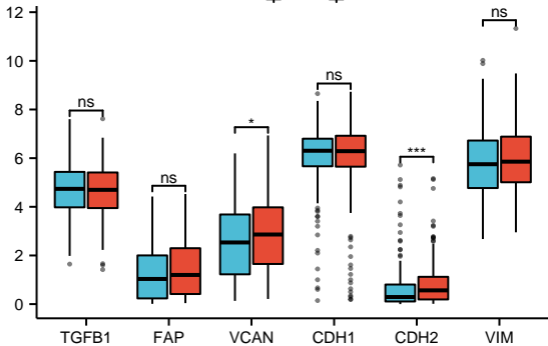

Supplement: Supplementary file 10 — Additional file 10: (A) BLCA with lymphovascular invasion expressed more FAP and VCAN than without lymphovascular invasion. In OS (B) and DSS (C) events, FAP, VCAN, N-cadherin, and Vimentin expression in the deceased group is higher than in the alive group. (D) In PFI events, VCAN and N-cadherin are more highly expressed in the deceased group. Immunohistochemistry (E) and dot blot (F) shows that the expression of TGF-β1, FAP, VCAN, N-cadherin, and Vimentin in BLCA tissues is significantly higher than in adjacent normal tissues, while the expression of E-cadherin in BLCA tissues is lower than in adjacent normal tissues. (G) TGF-β1 dominates stromal fibroblast-mediated EMT of bladder cancer cells via the FAP/VCAN axis to promote the invasion and metastasis of BLCA. [file 12967_2023_4303_MOESM10_ESM.zip › Additional file10D.pdf]

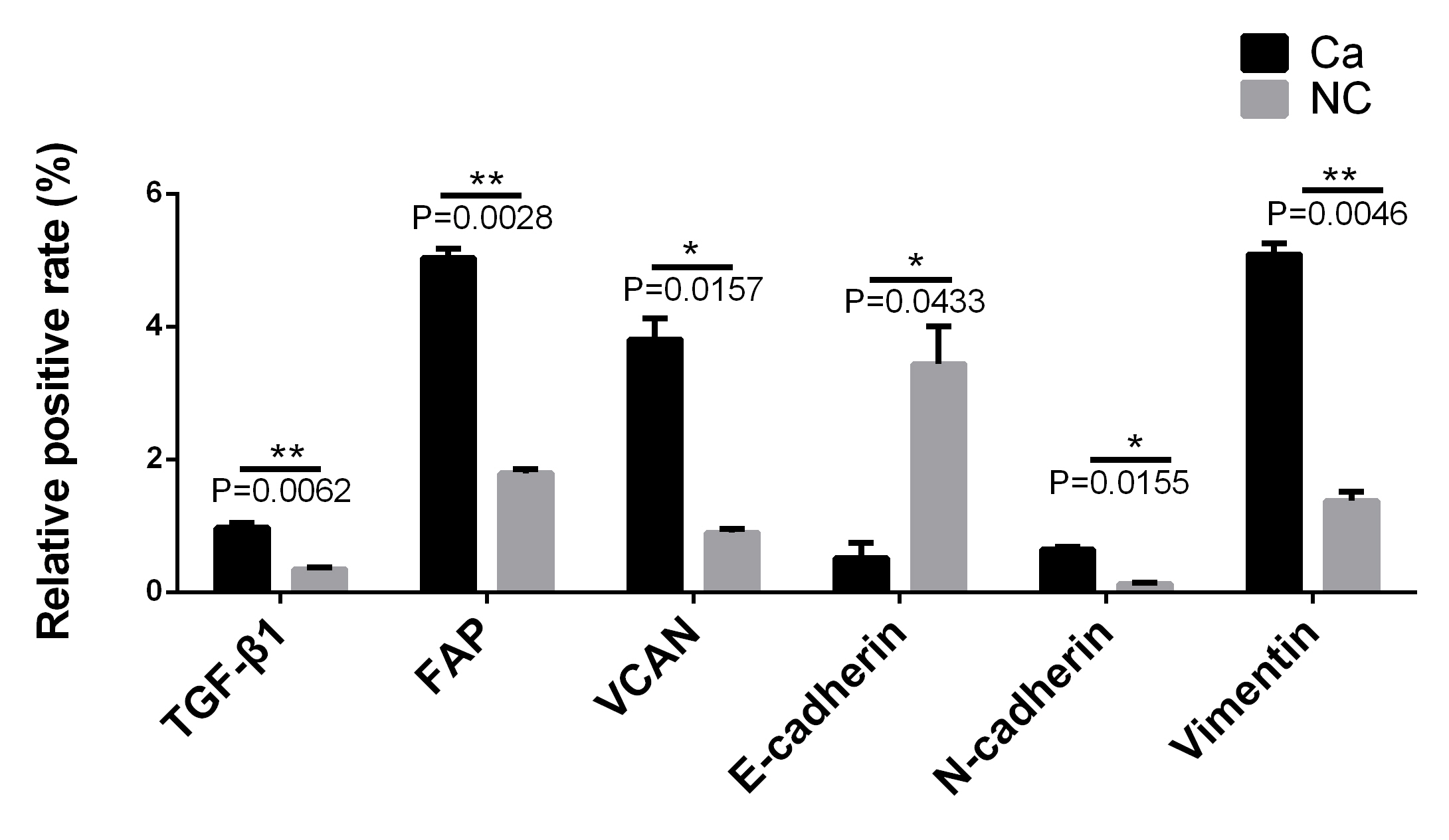

Supplement: Supplementary file 10 — Additional file 10: (A) BLCA with lymphovascular invasion expressed more FAP and VCAN than without lymphovascular invasion. In OS (B) and DSS (C) events, FAP, VCAN, N-cadherin, and Vimentin expression in the deceased group is higher than in the alive group. (D) In PFI events, VCAN and N-cadherin are more highly expressed in the deceased group. Immunohistochemistry (E) and dot blot (F) shows that the expression of TGF-β1, FAP, VCAN, N-cadherin, and Vimentin in BLCA tissues is significantly higher than in adjacent normal tissues, while the expression of E-cadherin in BLCA tissues is lower than in adjacent normal tissues. (G) TGF-β1 dominates stromal fibroblast-mediated EMT of bladder cancer cells via the FAP/VCAN axis to promote the invasion and metastasis of BLCA. [file 12967_2023_4303_MOESM10_ESM.zip › Additional file10E.jpg]

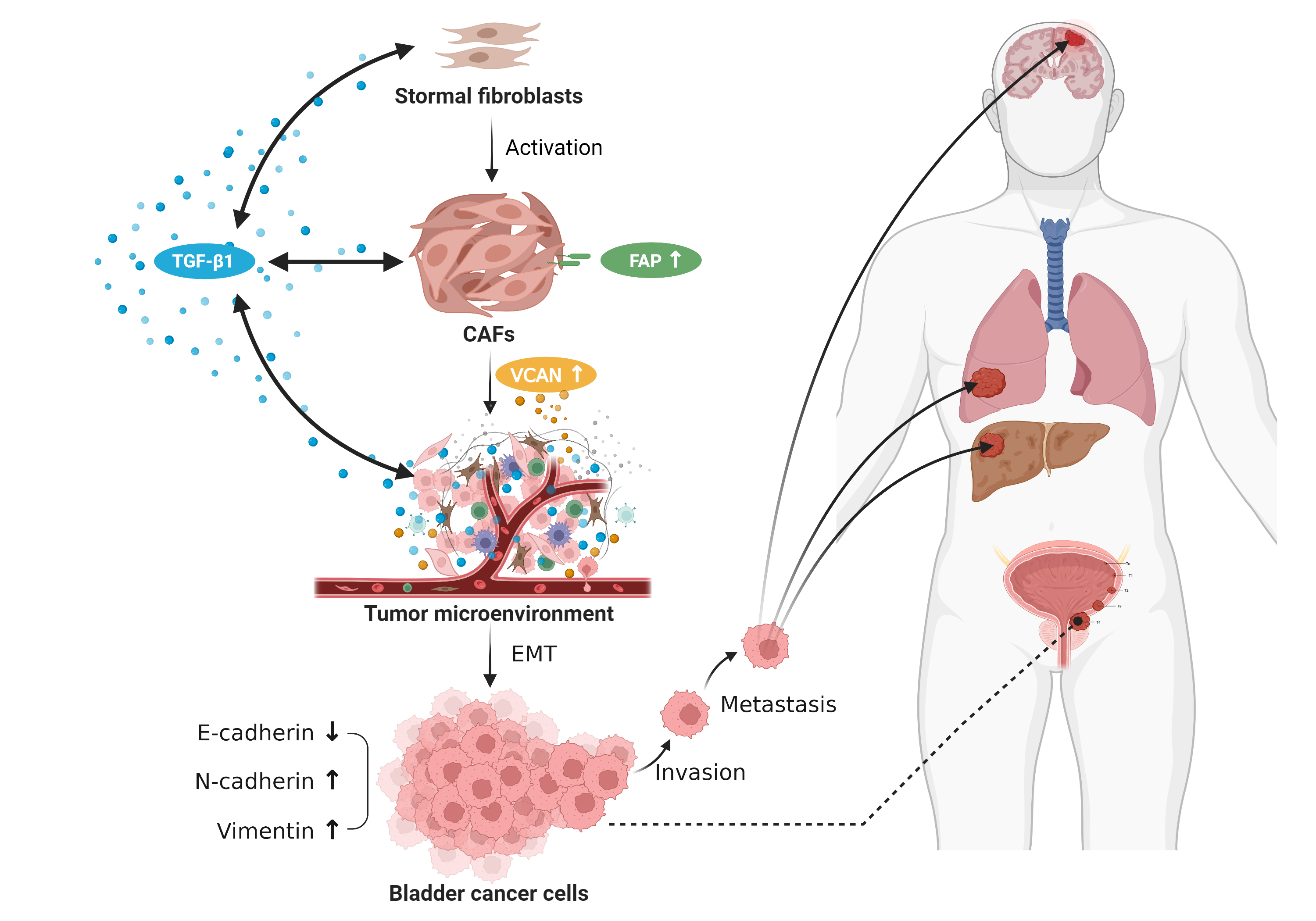

Supplement: Supplementary file 10 — Additional file 10: (A) BLCA with lymphovascular invasion expressed more FAP and VCAN than without lymphovascular invasion. In OS (B) and DSS (C) events, FAP, VCAN, N-cadherin, and Vimentin expression in the deceased group is higher than in the alive group. (D) In PFI events, VCAN and N-cadherin are more highly expressed in the deceased group. Immunohistochemistry (E) and dot blot (F) shows that the expression of TGF-β1, FAP, VCAN, N-cadherin, and Vimentin in BLCA tissues is significantly higher than in adjacent normal tissues, while the expression of E-cadherin in BLCA tissues is lower than in adjacent normal tissues. (G) TGF-β1 dominates stromal fibroblast-mediated EMT of bladder cancer cells via the FAP/VCAN axis to promote the invasion and metastasis of BLCA. [file 12967_2023_4303_MOESM10_ESM.zip › Additional file10G.png]
